# The Interaction of HLA-C1/KIR2DL2/L3 Promoted KIR2DL2/L3 Single-Positive/NKG2C-Positive Natural Killer Cell Reconstitution, Raising the Incidence of aGVHD after Hematopoietic Stem Cell Transplantation

**DOI:** 10.3389/fimmu.2022.814334

**Published:** 2022-04-29

**Authors:** Wei Zuo, Xing-Xing Yu, Xue-Fei Liu, Ying-Jun Chang, Yu Wang, Xiao-Hui Zhang, Lan-Ping Xu, Kai-Yan Liu, Xiao-Su Zhao, Xiao-Jun Huang, Xiang-Yu Zhao

**Affiliations:** Peking University People’s Hospital, Peking University Institute of Hematology, National Clinical Research Center for Hematologic Disease, Beijing Key Laboratory of Hematopoietic Stem Cell Transplantation, Beijing, China

**Keywords:** NK reconstitution, HLA-C (C1/C2), HSCT, NK receptor, KIR2DL2

## Abstract

NKG2C+ natural killer (NK) cell plays a vital role in CMV infection control after hematopoietic stem cell transplantation (HSCT). However, the modulation on NKG2C+ NK cell reconstitution is still unclear. NK cell education is affected by the interactions of HLA-I/killer immunoglobulin receptor (KIR). Our aim is to figure out which HLA-I/KIR interaction plays a dominant role in NKG2C+ NK education. Based on allogeneic haploidentical HSCT, we investigated the expansion and function of single KIR positive NKG2C+ NK cells *via* the interaction of KIR with both donor HLA and recipient HLA at days 30, 90, and 180 after HSCT. KIR2DL2/L3 single-positive/NKG2C^+^ cells were significantly expanded compared with KIR2DL1 or KIR3DL1 single-positive/NKG2C^+^ cells when donors and recipients were both HLA-C1/C1 or HLA-C1C1BW4 (*p* < 0.05), with higher NKp30 expression (*p* < 0.05). Moreover, the proportion of single KIR positive NK cells increased in both NKG2C^+^/NKG2A^-^ NK cells and conventional NKG2C^-^/NKG2A^-^ NK cells over time. We also observed that increased proportion of KIR2DL2/L3 single-positive/NKG2C^+^ NK cells correlated with higher incidence of acute graft-versus-host disease (aGVHD). Our study allows a better understanding of HLA-I/KIR interaction in the NKG2C+ NK cell education after HSCT.

## Introduction

Natural killer (NK) cells are lymphocytes involved in innate immunity that respond to viral infections and tumor cells; they are the first reconstituting lymphocytes after stem cell transplantation (1) and are related to clinical outcomes after hematopoietic stem cell transplantation (HSCT) (2). For NK cells to be functional, they must acquire inhibitory receptors that recognize self-HLA-I (3), which makes them tolerant of healthy cells but responsive to unhealthy cells with reduced expression of HLA class I, a process called “NK cell education” or “NK cell licensing” (3–6). Antibody-independent cytotoxicity of NK cells was activated by an imbalance between activating and inhibitory signals from the interactions between NK cell receptors and their ligands (7, 8), including the killer immunoglobulin-like receptors (KIRs), natural cytotoxicity receptors (NCRs), and Natural Killer Group (NKG)2x receptors (9–12). NK cell education was mainly affected by interaction of HLA-E/NKG2A and KIR/HLA (13–15). HLA-E polymorphism has an impact on relapse and disease-free survival in patients after HSCT (16, 17). It is revealed that NKG2A/CD94+ NK cells generated HSCT, which are blocked at an immature state with impaired functioning, and also have a potential negative impact on immune responsiveness and transplantation outcome (15, 18). Moreover, the upregulated interaction of HLA-E/NKG2A after HSCT was associated with poorer NK cytolysis and higher transplantation-related mortality (19, 20). Although NKG2A/HLA-E can also affect NK education and clinical outcomes (15, 16, 21), this study mainly focused on KIR/HLA-I interaction on NKG2C+ NK cells, which might be biased and not all-inclusive.

During NK cell education, NK subset expansion is tuned to be commensurate with the number of self-inhibitory receptors as well as by the presence of HLA ligand (22). Additionally, the expression of KIR receptors is influenced by extensive sequence polymorphisms of KIR genes (23), and promoter polymorphisms are obvious modifiers of transcription (24), altering expression (25). In healthy people, co-expression of HLA-C2 diminishes the cell surface density of KIR2DL1 on NK cells instead of impacting the respective frequencies of KIR2DL1-expressing cells within the NK repertoire (6, 26–28). On the contrary, another study found that individuals with KIR2DL1 or KIR3DL1 have greater numbers of NK cells expressing these genes if the HLA-C2 or HLA-Bw4 ligands are, respectively, present in the individuals (25, 29). Moreover, HLA-C1 ligands also affect the size of a subset of KIR2DL2/L3^+^ NK cells (30). Additionally, in the setting of allogeneic transplantation, NK reconstitution is affected by the HLA of recipients and/or donors. Our previous study demonstrated that when both donors and hosts presented all KIR ligands (HLA-C1, HLA-C2, and HLA-Bw4) and NK cells expressed 3 inhibitory receptors (KIR2DL1, KIR2DL2/L3, and KIR3DL1), the reconstituted NK cells achieved better education and contributed to the lowest rate of relapse among the patients (31, 32).

In addition, different KIR/HLA-I interactions can act through NK cell licensing to generate different levels of intrinsic responsiveness. The “Rheostat model” predicted weaker licensing by HLA-C1/KIR2DL2/L3 interactions than HLA-C2/KIR2DL1 (33, 34). Another study revealed that KIR2DL2/L3 and KIR2DL1 have similar capacities to licence NK cells (35). Therefore, the effect of the affinity of existing HLA-C ligands to cognate KIRs on NK licensing is still unclear, suggesting that the inhibitory signal strength and amount of available HLA-C ligands both influence NK cell education.

NKG2C is an activating receptor on the surface of NK cells that binds to antigen peptides presented by HLA-E molecules on the surface of target cells to activate NK cell functions (1, 21, 36–38). Recent studies have shown that NKG2C^+^ NK cells expanded distinctively with enhanced effector functions when stimulated by cytomegalovirus. These NK cells adapt to a pathogen and deliver a more potent recall response, called adaptive NK cells (4, 6, 39). NKG2C and the KIR family may participate in the NKG2C^+^ NK cell response. In human cytomegalovirus (HCMV)-seropositive patients, only NKG2C^+^ NK cells expressing KIR that recognized self-MHC ligands are hyperresponsive and make IFN-γ (40), suggesting that NKG2C^+^ NK cells are composed mainly of educated NK cells.

Obvious NKG2C^+^ NK cell expansions tend to be dominated by a single KIR clone, which is directed towards HLA-C-specific KIR encoded by the centromeric part of group A (CenA) haplotypes, but the moderately expanded NKG2C^+^ NK cells are often polyclonal (41). Group A haplotype encoded inhibitory KIR for all three major HLA class I, such as KIR2DL1 and KIR2DL3 on the centromeric part, specific for the HLA-C2 and -C1 epitopes, and HLA-Bw4 on the telomeric part, specific for KIR3DL1 (41, 42). A study of a cohort of HCMV serum-positive patients revealed that the expression of KIR2DL1 on NKG2C^+^ NK cells led to significantly larger clonal expansion than KIR2DL2, suggesting that an interaction of KIR2DL1 and HLA-C2 ligands seems to promote the expansion of NKG2C^+^ NK cells in response to HCMV infection (41). Another cohort of patients with HCMV infection post-transplantation was found to have expansion of NKG2C^+^ NK cells accompanied by co-expression of KIR with a strong bias towards KIR2DL2/L3; moreover, none of these patients expressed HLA-C1 as their only self-ligand (43). These studies revealed that under HCMV infection, the expansion of NKG2C^+^ NK cells showed a bias towards KIR2DL1 or KIR2DL2 expression due to the interaction between HLA-C and KIRs. However, previous studies did not focus on single KIR-positive NK cells without eliminating the interference of the interaction of other KIRs/ligands. In the post-transplantation setting, the interaction of donor and recipient KIR ligands in NKG2C^+^ NK cell education is still not clear.

Based on the haploidentical transplantation setting, we explored NKG2C^+^ NK cell reconstitution among patients post transplantation and creatively studied the proportion of single-positive KIR NK cells among the NKG2C^+^ NK cells to exclude the interference of other KIRs. Additionally, we also explored whether the number and type of KIRs (KIR2DL2/L3, KIR2DL1, and KIR3DL1) affect NKG2C^+^ expansion and, whether under different donor/recipient HLA types, the expansion, cytotoxicity function, and key molecules of single-positive KIR NK cells vary among NKG2C^+^ NK cells. We observed that KIR2DL2/L3 single NK cells were significantly expanded among NKG2C^+^ NK cells compared with KIR2DL1 and KIR3DL1. Furthermore, we investigated whether the genotype of *NKG2C* would affect KIR2DL2/L3 single KIR NK cell expansion among NKG2C^+^ NK cells and whether it would be related to the clinical outcomes of HCMV reactivation.

## Methods

### Patients

The prospective cohort included 128 patients with malignant hematologic disease who underwent HSCT at Peking University People’s Hospital between May 2016 and April 2017. The patient characteristics of the cohort are described in **Table 1**. All patients and donors provided written informed consent. The individuals of the cohort, who met the criterion that both recipients and donors were HCMV sero-positive before transplantation and recipients are HCMV-reactivated post allo-HSCT, were defined as “HCMV-reactivating” individuals. We also described the HLA-C status of the patients in cohort in **Table 2**.The flow chart of cohort enrolment is presented in **Supplementary Figure 5**. This study was approved by the Institutional Review Committee of the Institute of Haematology, Peking University, and it was registered at www.clinicaltrials.gov (NCT02978274). Quantitative and qualitative reconstructions of NK cells were performed at 30, 90, and 180 days after transplantation.

**Table 1:** Patient characteristics.

Characteristic	Group 1(C1C1)	Group 2(C1BW4)	Group 3(C1C2BW4)	Total	P
**No. of patients**	18	11	19	128	
**Patient median age (range), years**	30.5(16–58)	37(20–56)	29(18–56)	33.5(15–63)	0.7056
**Patient sex, male, No. (%)**	12(66.67%)	9(81.8%)	10(52.63%)	82(64.06%)	0.2659
**Donor median age (range), years**	43(14–60)	41(19–54)	40(14–77)	31(8–71)	0.6908
**Donor sex, male, No. (%)**	14(77.78%)	9(81.82%)	18(94.74%)	102(79.69%)	0.3194
**Donor-recipient sex, No. (%)**					0.3425
Male to male	10(55.56%)	6(54.55%)	10(52.63%)	63(49.22%)	
Male to female	3(16.67%)	3(27.27%)	8(42.11%)	33(25.78%)	
Female to male	3(16.67%)	2(18.18%)	0(0.00%)	19(14.84%)	
Female to female	2(11.11%)	0(0.00%)	1(5.26%)	13(10.16%)	
**NKG2C genotype**					0.3027
*NKG2C^wt/wt^*	6(33.33%)	4(36.36%)	11(57.89%)	79(61.72%)	
*NKG2C^wt/del^*	11(61.11%)	7(63.64%)	6(31.58%)	44(34.38%)	
*NKG2C^del/del^*	1(5.56%)	0(0.00%)	2(10.53%)	5(3.91%)	
**Diagnosis, No. (%)**					0.2870
AML	11(61.11%)	8(72.73%)	10(52.63%)	63(49.22%)	
CML	0(0.00%)	0(0.00%)	3(15.79%)	7(5.47%)	
ALL	6(33.33%)	2(18.18%)	3(15.79%)	38(29.69%)	
MDS	1(5.56%)	1(9.09%)	3(15.79%)	20(15.63%)	
**ABO matched grafts, No. (%)**					0.1035
Matched	9(50.00%)	10(90.91%)	14(73.68%)	80(62.50%)	
Major mismatch	5(27.78%)	1(9.09%)	1(5.26%)	29(22.66%)	
Minor mismatch	2(11.11%)	0(0.00%)	4(21.05%)	20(15.63%)	
**aGVHD, No. (%)**	8(44.44%)	7(63.64%)	12(63.16%)	73(57.03%)	0.4423
**Donor KIR haplotype**	(n=13)	(n=9)	(n=16)	(n=111)	0.3656
A/A, **No.** (%)	9(69.23%)	8(88.89%)	14(87.50%)	94(84.68%)	
A/B, **No.** (%)	4(30.77%)	1(11.11%)	2(12.50%)	17(15.32%)	
**Recipient sero-positive**, **No.(%)**	18(100.00%)	11(100%)	19(100.00%)	124(96.88%)	
**Donor sero-positive**, **No. (%)**	17(94.44%)	11(100%)	18(94.74%)	117(91.41%)	0.7326
**CMV status after HSCT**					
Reactivation, **No.** (%)	16(88.89%)	10(90.91%)	17(89.47%)	115(89.84%)	0.9850
Refractory, **No.** (%)	12(66.67%)	10(90.91%)	12(63.16%)	88(68.75%)	0.2418
**“CMV reactivating”**, **No.** (%)	15(83.33%)	10(90.91%)	17(89.47%)	105(82.03%)	0.7905
**Clinical outcomes**					
Relapse, **No.** (%)	2(11.11%)	2(18.18%)	2(10.53%)	16(12.50%)	0.8089
Death, **No.** (%)	2(11.11%)	2(18.18%)	3(15.79%)	14(10.94%)	0.8561
**Duration of CMV viremia (days, median)**	14	23	17	15	0.5428
**the timing of CMV reactivation after HSCT (days, median)**	37	36	34	37	0.932

AML, acute myelocytic leukaemia; CML, chronic myeloid leukaemia; ALL, acute lymphoid leukaemia; MDS, myelodysplastic syndrome; HCMV, human cytomegalovirus; aGVHD, acute graft-versus-host disease. Refractory reactivation was defined as CMV reactivation lasting over 2 weeks. CMV-reactivating individuals were defined as recipients, and their donors were both CMV sero-positive before transplantation. Recipients were HCMV virus-positive post allo-HSCT. Only some individuals in the cohort had follow-up data on the KIR genotype. The numbers of patients with “CMV reactivation” in groups 1, 2, and 3 were 10, 9, and 12 on Day30; 14, 9, and 15 on Day90; and 14, 8, and 13 on Day180, respectively. Here, we show the characteristics of patients in the whole cohort.

**Table 2:** Patient HLA-C status.

HLA	Recipient	Donor	Recipient–Donor
C1C1 (*n*, %)	39	30	18
C2C2 (*n*, %)	3	2	0
C1C1BW4 (*n*, %)	36	29	11
C2C2BW4 (*n*, %)	7	9	1
C1C2 (*n*, %)	8	16	4
C1C2BW4 (*n*, %)	35	42	19

We showed the HLA-C status of recipients and donors who had the same HLA-C as their corresponding donors.

### Transplants

All patients received myeloablative regimens (44, 45). Pretransplant conditioning was performed with cytarabine (4 g/m^2^ per day on Days −8 to −9), busulfan (3.2 mg/kg per day IV on Days −8 to −6), cyclophosphamide (1.8 g/m^2^ per day on Days −5 to −4), semustine (250 mg/m^2^ on Day −3), and rabbit anti-thymocyte globulin (thymoglobulin; Imtix Sangstat, Lyon, France; 2.5 mg/kg per day IV on Days −5 to −2) (44, 46). GVHD prophylaxis was performed with cyclosporine, mycophenolate mofetil, and short-course methotrexate, as previously described (44, 46). The transplant procedure was performed as previously described (31, 47, 48).

### HLA and KIR Typing

Patient and donor DNA was prepared from peripheral blood mononuclear cells (PBMCs) from fresh blood samples for pretransplant HLA typing and stored at −40°C. Molecular HLA typing and KIR genotyping were performed (One Lambda, Canoga Park, CA) in accordance with the manufacturer’s instructions. HLA-C and HLA-B alleles were identified with high-resolution DNA-based HLA typing and segregated into the following epitope groups: HLA-C group 1 (HLA-CAsn80: HLA-Cw1, 3, 7, 8, 13, and 14 alleles, ligand of KIR2DL2/L3), HLA-C group 2 (HLA-CLys80: HLA-Cw2, 4, 5, 6, 12, 15, 17, and 18 alleles, ligand of KIR2DL1), and HLA-Bw4 (ligand of KIR3DL1). Chimerism analyses were based on a report by Qin et al. (Shanghai Tissuebank Diagnostics, Shanghai, China) (47). Complete donor chimerism was defined as 100% donor short tandem repeat sequencing. The centromeric part of group A (cenA) haplotypes encoded C2-specific KIR2DL1 and C1-specific KIR2DL3. All non-A haplotypes on the centromeric side are referred to as group B haplotypes, encoding KIR2DL2 that recognized C1.

### Monitoring for HCMV Reactivation

A real-time polymerase chain reaction (PCR) kit from Shenzhen PG Biotechnology Co., Ltd., China was used to detect HCMV reactivation through plasma HCMV DNA detection twice a week. HCMV infection was diagnosed according to previously published criteria (49–51), which means HCMV DNA ≥ 1 × 10^3^ copies/ml in peripheral blood based on the HCMV PCR assay. Refractory reactivation was defined as HCMV reactivation lasting over 2 weeks (51, 52).

### *NKG2C* Genotyping

Donor DNA was isolated from total fresh blood samples using the Puregene Blood Core kit B (Qiagen), and *NKG2C* zygosity was assessed as previously described (53). The patients were divided into *NKG2C^del/del^*, *NKG2C^wt/wt^*, and *NKG2C^wt/del^* groups according to the donor genotype.

### Flow Cytometry Analyses

Surface marker staining of PBMCs from patients’ fresh blood samples for the following markers was performed in PBS at 4°C for 30 min following Fc blockade: CD3 (BD, UCHT1), CD56 (BD, NKCM16), CD335 (NKp46) (BD, 9E2/NKp46), CD337 (NKp30) (BD, p30-15), CD57 (BD, NK-1), DNAM1 (BD, DX11), NKG2C (CD159c) (Miltenyi Biotec, REA205), NKG2A (CD159a) (Miltenyi Biotec, REA110), NKG2D (BD, 1D11), KIR2DL1 (BD, HP-3E4), KIR2DL2/L3 (Beckman Coulter), KIR3DL1 (Miltenyi Biotec, REA168), CD107a (BD, H4A3), and IFN-γ (BioLegend, B27). Acquisition was performed on an LSR Fortessa instrument (BD Biosciences), and Flowjo software (TreeStar) was used for analysis. Single-stain controls for compensation were generated utilizing UltraComp eBeads (eBioscience, BioLegend, and Miltenyi Biotec), and the analysis of acquired samples was performed in FlowJo (FlowJo, LLC, Ashland, CA) data analysis software. Data were gated using FlowJo (FlowJo, LLC). Following data acquisition, the channel intensity was normalized using calibration beads. Before the experiment, we verified the configuration by selecting an appropriate configuration and defining a new baseline every 24 h. We set up and ran a performance check. The anti-human monoclonal antibodies used for flow cytometry are listed in **Supplementary Table 1**.

### Cytotoxicity Assays

The cytotoxicity test used cryopreserved PBMC samples from patients. Two million freshly thawed PBMCs were washed twice and transferred to a 96-well round-bottom plate (Corning) with RPMI 1640 supplemented with 10% fetal calf serum and 1,000 IU/ml interleukin 2 (IL-2; Beijing Double-Crane Pharmaceutical Co., Ltd.) for 10–14 h. PBMCs were cultured in preparation for both spontaneous and IL-2-stimulated NK cytotoxicity assays. The cytotoxicity and cytokine secretion of the NK cells was determined by CD107a and IFN-γ expression against the target K562 cells at an effector-to-target ratio of 5:1 for 4 h, with GolgiStop (0.7 µl/ml, BD Biosciences) added after 1 h to trap proteins in the cytoplasm. After fixation and permeabilization of PBMCs using a Pharmingen Intracellular Staining kit (BD Pharmingen, San Diego, CA, USA) for 40 min at 4°C, we washed it twice and added the IFN-γ antibody.

### Statistical Analysis

Continuous variables were compared using a nonparametric test (Mann–Whitney *U* test for two groups and Kruskal–Wallis *H* test for multiple groups) and parametric tests (two-sample *t*-test, paired *t*-test, and one-way analysis of variance). The association between the proportion of KIR2DL2/L3 single-positive/NKG2C^+^ NK cells and clinical outcomes and HCMV reactivation type was analyzed by the chi-square test. ROC curve was used to get the cutoff value. We used Cox regression analysis to perform univariate and multivariate analyses. Variables with *p* < 0.10 in univariate analysis were further included in the multivariate analysis. The cumulative incidence of acute graft-versus-host disease (aGVHD) was estimated using the competing risk model by R software 4.1.1. Statistical analyses were performed using SPSS 26.0 and GraphPad Prism 8.0. *p*-values < 0.05 were considered statistically significant.

## Results

### The Number and Type of KIR Ligands Shared by Donors and Recipients Have No Effect on NKG2C Expansion and Reactivity

To determine whether the number and types of KIR ligands influence NKG2C^+^ NK cell expansion, phenotype, or cytotoxicity function, we divided the patients into 4 groups (groups W, X, Y, and Z) according to the number of KIR ligands shared by donors and recipients, and divided the patients into 6 groups (group A to group F) according to the types of KIR ligand presented by both donor and recipient. The proportion of NKG2C^+^ NK cells among the NK cells, the absolute number of NKG2C^+^ NK cells, and the expression of NKp30, NKp46, and DNAM-1 and secretion of IFN-γ and CD107a by NK cells were analyzed among groups W to Z and among groups A to F.

**Figure 1:**
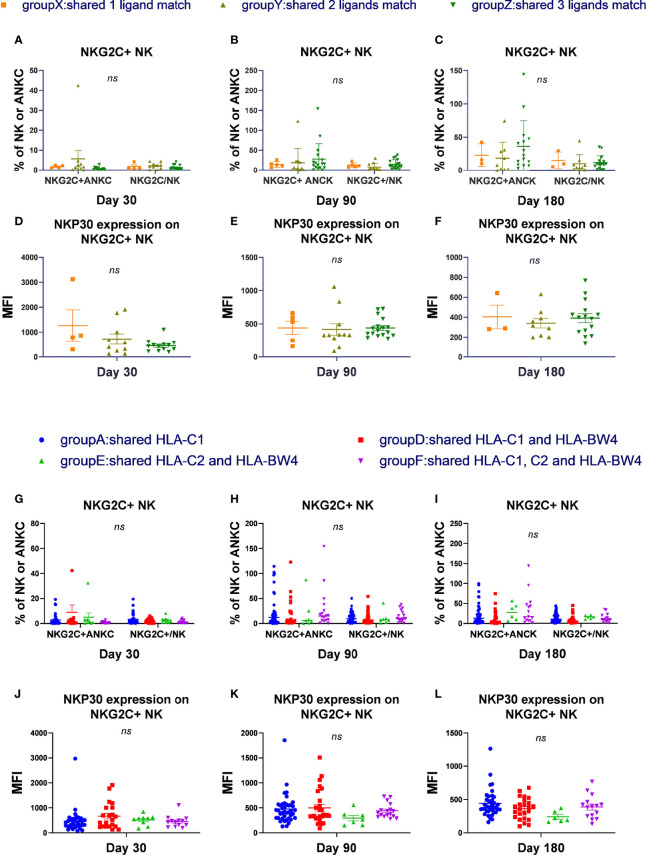
The number and types of KIR ligands shared by donors and recipients has no effect on NKG2C expansion and reactivity. According to the number of HLA-C donors and recipients both shared, we divided the patients into 4 groups, group W to group Z and excluded group W. According to the types of matches of donor-patient HLA-C, we divided all the patients in the cohort into 6 groups and excluded groups B and C. **(A–C)** The proportion of NKG2C+ NK cells among NK cells and the absolute number of NKG2C+ NK cells (ANKC, absolute NK count) from patients after HSCT in group X (day 30, *n* = 4; day 90, *n* = 5; day 180, *n* = 3), group Y (day 30, *n* = 10; day 90, *n* = 11; day 180, *n* = 9), and group Z (day 30, *n* = 13; day 90, *n* = 17; day 180, *n* = 15). **(G–I)** The proportion of NKG2C+ NK cells among NK cells and the absolute number of NKG2C^+^ NK cells (ANKC, absolute NK count) from patients after HSCT in group A (day 30, *n* = 35; day 90, *n* = 40; day 180, *n* = 38), group D (day 30, *n* = 21; day 90, *n* = 28; day 180, *n* = 24), group E (day 30, *n* = 9, day 90, *n* = 7, day 180, *n* = 6), and group F (day 30, *n* = 13, day 90, *n* = 17; day 180, *n* = 15). **(D–F, J–L)** The mean fluorescence intensity (MFI) of NKp30 on NKG2C^+^/NKG2A^-^ NK cells in groups X to Z and groups A, D, E, and F on days 30, 90, and 180. Each plot (triangle, circle, or square) shows one patient with the indicated receptor expression. The middle horizontal lines show the mean values for each group, and the upper and lower horizontal lines show the standard error of the mean. n.s., not significant (Kruskal–Wallis *H* test).

Group W included donors and recipients who were C2C2BW4×C1C1 or C1C1BW4×C2C2, which shared a 0 ligand matches; group X included donors and recipients who were C1C1BW4×C2C1 or C2C2BW4×C2C1, which shared 1 ligand match; group Y included donors and recipients who were C1C1BW4×C1C1BW4, C2C2BW4×C2C2BW4, or C1C2×C1C2, which shared 2 ligand matches; and group Z included donors and recipients who were C1C2BW4×C1C2BW4, which shared 3 ligand matches. Group W did not undergo statistical analysis because their sample sizes were too small (**Figures 1A–F**).

In group A, donors and recipients both presented HLA-C1, where donor recipients were C1C1×C1C1, C1C1×C1C1BW4, or C1C1×C1C2. In group B, donors and recipients both presented HLA-C2, where donor recipients were C2C2×C2C2, C2C2×C2BW4, or C2C2×C1C2. In group C, donors and recipients both presented HLA-BW4, and were C1C1BW4×C2BW4. In group D, donors and recipients both presented HLA-C1 and HLA-BW4, and donor recipients were C1C1BW4×C1C1BW4 or C1C1BW4×C1C2BW4. In group E, donors and recipients both presented HLA-C2 and HLA-BW4, and donor recipients were C2C2BW4×C2C2BW4 or C2C2BW4-C1C2BW4. In group F, donors and recipients both presented HLA-C1, C2, and HLA-BW4, and were both C1C2BW4. Group B and group C did not undergo statistical analysis because their sample sizes were too small (**Figures 1G–L**).

In the cohort and in CMV-reactivated individuals, we found that there were no obvious differences among groups X, Y, and Z and among groups A, D, E, and F, regarding the proportion of NKG2C^+^ NK cells among the NK cells or the absolute number of NKG2C^+^ NK cells (cells/μl) on days 30, 90, and 180. Moreover, the expression of NKp30, NKP46, and DNAM-1 and the cytotoxicity function measured by IFN-γ and CD107a were comparable among groups X to Z and among groups A, D, E, and F on days 30, 90, and 180. These results indicated that the number and types of KIR ligands shared by donors and recipients were irrelevant to NKG2C^+^ expansion, expression of receptors, and cytotoxicity during NK cell education.

### The Presence of HLA-C1 Enhanced the Expansion of KIR2DL2/3 Single-Positive NKG2C^+^ NK Cells, With Increased Expression of NKp30

To evaluate whether the HLA-C/KIR interaction will affect the expansion of NK subsets, according to the combination of donor–recipient KIR ligands, we divided the patients into 3 groups, where the donor and recipient were both C1C1 in group 1, C1C1BW4 in group 2, and C1C2BW4 in group 3. We analyzed the percentage of the three NK cell populations expressing 1 specific KIR among the NKG2C^+^ NK cells and the expression levels of NKp30, NKP46, and DNAM-1. Gating strategy was described in **Supplementary Figure 1**.

**Figure 2:**
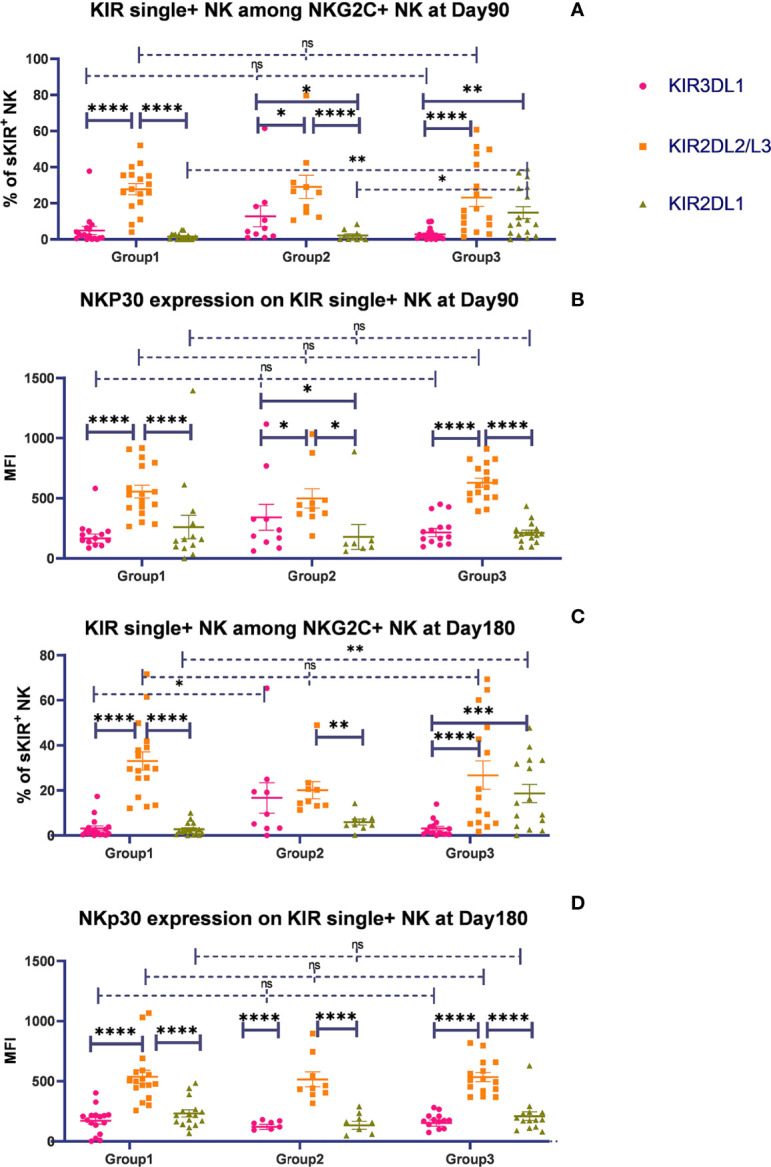
The presence of HLA-C1 enhanced the expansion of KIR2DL2/3 single-positive NKG2C^+^ NK cells and the expression of NKp30. The surface markers of peripheral blood mononuclear cells (PBMCs) from patients’ fresh blood sample were analyzed by flow cytometry, performed in PBS at 4°C for 30 min following Fc blockade. **(A, C)** The proportion of KIR2DL1, KIR2DL2/L3, and KIR3DL1 single-positive NKG2C^+^/NKG2A^-^/CD56^+^ NK cells from patients after HSCT in group 1 (recipient donor C1C1; day 90, *n* = 17; day 180, *n* = 17), group 2 (recipient donor C1BW4; day 90, *n* = 10; day 180, *n* = 9), and group 3 (recipient donor C1C2BW4; day 90, *n* = 17; day 180, *n* = 15), with the presence of their respective ligands. **(B, D)** The mean fluorescence intensity (MFI) of NKp30 on KIR2DL1, KIR2DL2/L3, and KIR3DL1 single-positive NKG2C^+^/NKG2A^-^/CD56^+^ NK cells from patients after HSCT in group 1 (day 90, *n* = 17; day 180, *n* = 17), group 2 (day 90, *n* = 10; day 180, *n* = 9), and group 3 (day 90, *n* = 17; day 180, *n* = 15). Each plot (triangle, circle, or square) represents one patient with the indicated receptor expression. The middle horizontal lines show the mean values for each group, and the upper and lower horizontal lines show the standard error of the mean. *p < 0.05; **p < 0.01; ***p < 0.001; ****p < 0.0001; n.s., not significant (Kruskal–Wallis *H* test).

On days 90 and 180, in the whole cohort, the proportion of KIR2DL2/L3 single-positive/NKG2C^+^ NK cells among the NKG2C^+^ NK cells was higher than those of KIR2DL1 and KIR3DL1, in group 1 (**Table 3**), which suggested that the existence of a combination of HLA-C1 and KIR2DL2/L3 enhanced the KIR single-positive NK cell expansion (**Figures 2A, C**). However, in group 3, the proportions of KIR2DL2/L3 and KIR2DL1 single-positive/NKG2C^+^ cells were both higher than that of KIR3DL1 cells (**Table 3**), demonstrating that the coexistence of both donor and host HLA-C1 or HLA-C2 was conducive to stronger expansion of KIR single-positive NK cells than HLA-BW4 (**Figures 2A, C**). In addition, the MFI of NKp30 expression on KIR2DL2/L3 single-positive/NKG2C^+^ NK cells was significantly higher than that of the KIR2DL1 single-positive/NKG2C^+^ and KIR3DL1 single-positive/NKG2C^+^, in group 1 (**Figures A, C**), group 2, and group 3 (**Figures 2B, D**) (**Table 3**), which indicated that the combination of KIR2DL2/L3 single-positive/NKG2C^+^ and HLA-C1 significantly correlated with a higher expression level of NKp30.

**Table 3:** The proportion and the MFI of NKp30 expression of single KIR positive/NKG2C^+^ NK cells.

The proportion of single KIR-positive/NKG2C^+^ NK cells						
	Group 1 (C1C1-C1C1)		Group 2 (C1C1BW4-C1C1BW4)		Group 3 (C1C2BW4-C1C2BW4)	
90 days	KIR2DL2/3		KIR2DL2/3		KIR3DL1	
	27.76% ± 3.06%		29.15% ± 6.41%		2.75% ± 0.77%	
	vs. KIR2DL1	vs. KIR3DL1	vs. KIR2DL1	vs. KIR3DL1	vs. KIR2DL2/L3	vs. KIR2DL1
	1.58% ± 0.42%	4.93% ± 2.18%	2.11% ± 0.89%	12.80% ± 5.86%	23.11% ± 4.79%	14.80% ± 3.37%
	*p* < 0.0001	*p* < 0.0001	*p* < 0.0001	*p* = 0.0115	*p* < 0.0001	*p* = 0.0023
180 days	KIR2DL2/3		KIR2DL2/3		KIR3DL1	
	33.07% ± 4.01%		20.08% ± 3.85%		3.13% ± 0.96%	
	vs. KIR2DL1	vs. KIR3DL1	vs. KIR2DL1	vs. KIR3DL1	vs. KIR2DL2/L3	vs. KIR2DL1
	2.79% ± 0.68%	3.16% ± 1.09%	16.67% ± 6.74%	5.98% ± 1.29%	26.79% ± 6.32%	18.67% ± 4.06%
	*p* < 0.0001	*p* < 0.0001	*p* = 0.0003	0.2581	*p* < 0.0001	*p* = 0.0007
**The MFI of Nkp30 expression of single KIR-positive/NKG2C^+^ NK cells**						
90 days	KIR2DL2/3		KIR2DL2/3		KIR2DL2/L3	
	555.5 ± 52.47		499.2 ± 81.26		629.8 ± 37.33	
	vs. KIR2DL1	vs. KIR3DL1	vs. KIR2DL1	vs. KIR3DL1	vs. KIR2DL1	vs. KIR3DL1
	260.9 ± 98.02	165.8 ± 37.69	178.8 ± 104.5	341.7 ± 107.2	213.7 ± 21.81	215.4 ± 34.10
	*p* < 0.0001	*p* = 0.0138	*p* = 0.0044	*p* = 0.0272	*p* < 0.0001	*p* < 0.0001
180 days	KIR2DL2/3		KIR2DL2/3		KIR3DL1	
	537.2 ± 53.86		515.5 ± 62.53		534.7 ± 37.56	
	vs. KIR2DL1	vs. KIR3DL1	vs. KIR2DL1	vs. KIR3DL1	vs. KIR2DL1	vs. KIR3DL1
	232.4 ± 29.14	170.4 ± 27.39	134.7 ± 32.64	121.6 ± 20.89	209.2 ± 36.24	152.3 ± 25.83
	*p* < 0.0001	*p* < 0.0001	*p* < 0.0001	*p* < 0.0001	*p* < 0.0001	*p* = 0.0007

We additionally analyzed the expansion and function of single-positive NK cells in individuals in the cohort screened by the criteria that both recipients and donors are HCMV sero-positive before transplantation and recipients are HCMV virus-positive post allo-HSCT, called HCMV-reactivating individuals. In HCMV-reactivating individuals, on days 90 and 180, the proportion of KIR2DL2/L3 single-positive/NKG2C^+^ NK cells among NKG2C^+^ NK cells was consistently higher than that of KIR2DL1 and KIR3DL1 NK cells (data not shown), which suggested that HCMV infection might not affect the expansion of KIR2DL2/L3 single-positive/NKG2C^+^ NK cells.

These results further demonstrated that the integration of KIR2DL2/L3 single-positive/NKG2C^+^ and HLA-C1 cells mainly contributed to NKG2C^+^ NK cell education, including KIR single-positive NK cell expansion and activating receptor expression.

Additionally, KIR single-positive NK cells showed comparable functions of cytotoxicity. There were no significant differences in CD107a expression or IFN-γ secretion among KIR2DL1, KIR2DL2/L3, or KIR3DL1 single-positive NK cells against K562, on day 30 and 90 after HSCT in group 1 (day 30: CD107a: *p* = 0.482, IFN-γ: *p* = 0.124; day 90: CD107: *p* = 0.007, IFN-γ: *p* = 0.057), group 2 (day 30: CD107a: *p* = 0.075, IFN-γ: *p* = 0.151; day 90: CD107a: *p* = 0.155, IFN-γ: *p* = 0.841), and group 3 (day 30: CD107a: *p* = 0.394, IFN-γ: *p* = 0.523; day 90: CD107a: *p* = 0.925, IFN-γ: *p* = 0.234) (**Supplementary Figure 3**). The data indicated that three NK cell populations expressing 1 specific KIR had comparable reactivity. Similarly, in the HCMV-reactivating individuals, the same results were observed (data not shown).

We further analyzed the expansion of KIR2DL1, KIR2DL2/L3, or KIR3DL1 single-positive NK cells in another group, in which either the donor or recipient was C1. We observed that on days 30 (*p* = 0.186), 90 (*p* = 0.108), and 180 (*p* = 0.046), KIR2DL2/L3 single-positive NK cells tended to expand significantly more than KIR3DL1 single-positive NK cells (data not shown). This result suggested that even if only one side had HLA-C1, KIR2DL2/L3 single-positive NK cells might significantly proliferate.

### The Proportion of Single KIR Positive NK Cells Increased in Both NKG2C^+^/NKG2A^-^ NK Cells and Conventional NKG2C^-^/NKG2A^-^ NK Cells Over Time

**Figure 3:**
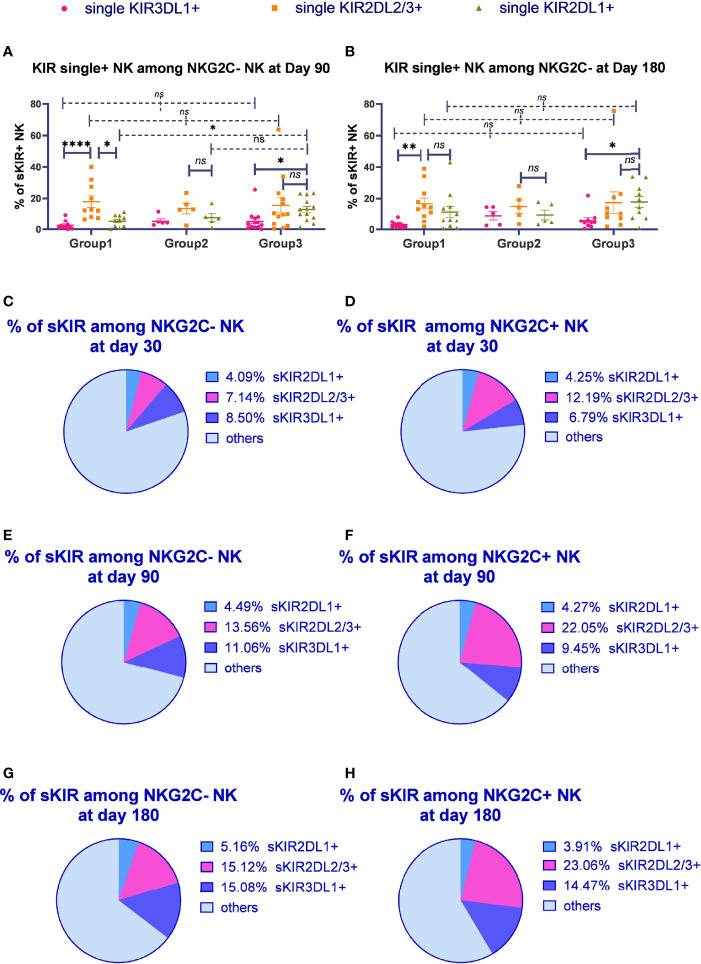
The proportion of single KIR positive NK cells increased in both NKG2C^+^/NKG2A^-^ NK cells and conventional NKG2C^-^/NKG2A^-^ NK cells over time. sKIR2DL1+, single KIR2DL1 positive; sKIR2DL2/L3+, single KIR2DL2/L3 positive; sKIR3DL1+, single KIR3DL1 positive. The surface markers of peripheral blood mononuclear cells (PBMCs) from patients’ fresh blood sample were analyzed by flow cytometry, performed in PBS at 4°C for 30 min following Fc blockade. **(A, B)** The proportion of KIR2DL2/L3 single-positive/NKG2C+ NK cells among the NKG2A- NK cells from the patients after HSCT with available data in group 1 (recipient-donor C1C1; day 90, *n* = 10; day 180, *n* = 11), group 2 (recipient-donor C1BW4; day 90, *n* = 5; day 180, *n* = 5), and group 3 (recipient-donor C1C2BW4; day 90, *n* = 13; day 180, *n* = 10). Each plot (triangle, circle, or square) represents one patient with the indicated receptor expression; middle horizontal lines show mean values for each group, and upper and lower horizontal lines show the standard error of the mean. *p < 0.05; **p < 0.01; n.s.: not significant (Kruskal–Wallis *H* test). **(C–H)** The mean proportion of single KIR NK cells among NKG2C- and NKG2C+ NK cells from patients after HSCT with available data in the whole cohort on days 30 (*n* = 54), 90 (*n* = 80), and 180 (*n* = 66).

To determine whether KIR2DL2/L3 single-positive/NKG2C^+^ NK cells characteristically expanded among NKG2C^+^ NK cells, we analyzed the proportion of KIR single-positive NK cells among the total CD56^dim^/NKG2A^-^ **/**NKG2C**^-^** conventional NK cells on days 90 and 180 after HSCT. Gating strategy was described in **Supplementary Figure 2**. In group 1 (C1C1-C1C1), the proportion of KIR2DL2/L3 single-positive NK cells among the CD56^dim^/NKG2A^-^ **/**NKG2C^-^ NK cells was significantly higher than that of KIR3DL1 on days 90 (*p* < 0.0001) and 180 (*p* = 0.001). Meanwhile, in group 2 (C1C1BW4-C1C1BW4), the expansion of KIR2DL2/L3, KIR2DL1, and KIR3DL1 was comparable on days 90 (*p* = 0.208) and 180 (*p* = 0.481) after HSCT. In group 3 (C1C2BW4-C1C2BW4), the proportion of KIR2DL1 single-positive NK cells among CD56^dim^/NKG2A^-^/NKG2C^-^ NK cells was significantly higher than that of KIR3DL1 single-positive NK cells on days 90 (*p* = 0.028) and 180 (*p* = 0.023) (**Figures 3A, B**).

We further observed the proportion of single-KIR-positive NK cells among CD56^dim^/NKG2A^-^/NKG2C^-^ and CD56^dim^/NKG2A^-^/NKG2C^+^ NK cells in the whole cohort (**Figures 3C–H**). The proportion of KIR2DL2/L3 single-positive NK cells among NKG2C^+^ NK cells significantly increased compared with that among NKG2C^-^ NK cells on days 30 (*p* = 0.012), 90 (*p* < 0.0001), and 180 (*p* < 0.0001), suggesting that KIR2DL2/L3 single-positive/NKG2C^+^ NK cells might mainly expand in the NKG2C^+^ NK cell pool in patients after HSCT.

Importantly, we noticed the relative increase for KIR2DL2/L3 single-positive NK cells as well as KIR3DL1 single-positive NK cells over time. Moreover, the proportion of single KIR positive NK cells, the sum of KIR2DL1, KIR2DL2/L3, and KIR3DL1, increased in both NKG2C- NK population and NKG2C+ NK population along with time (day 30: NKG2C^+^ = 22.23%, NKG2C^-^ = 19.73%; day 90: NKG2C^+^ = 35.78%, NKG2C^-^ = 29.11%; day 180: NKG2C^+^ = 41.44%, NKG2C^-^ = 35.36%).

### NKG2C Genotype Was Irrelevant With KIR2DL2/L3 Single-Positive/NKG2C^+^ NK Cells’ Significant Expansion

Considering that NKG2C expression on NK cells could be influenced by NKG2C genotyping (54, 55), we further analyzed the effect of donor NKG2C genotyping on KIR single-positive/NKG2C+ NK cell expansion. We found that in both the *NKG2C^wt/del^* and *NKG2C^wt/wt^* groups, the percentage of KIR2DL2/L3 single-positive/NKG2C^+^ NK cells among the NKG2C^+^ NK cells was significantly higher than single-KIR2DL1^+^/NKG2C^+^ and single-KIR3DL1^+^/NKG2C^+^ NK cells on days 90 and 180 in the whole cohort (**Supplementary Figures 4A–C**) and in HCMV-reactivating individuals (data not shown). We also found that the percentage of KIR2DL2/L3 single-positive/NKG2C^+^ NK cells among the NKG2C^+^ NK cells between the *NKG2C^wt/wt^* and the *NKG2C^wt/del^* groups were comparable in both the whole cohort and in HCMV-reactivating individuals, indicating that NKG2C genotype did not influence the expansion of KIR2DL2/L3 single-positive/NKG2C^+^ NK cells (**Supplementary Figures 4A–D**).

### KIR Typing Was Irrelevant to the Expansion of KIR2DL2/L3 Single-Positive/NKG2C^+^ NK Cells

We additionally analyzed the KIR typing and the expansion of KIR2DL2/L3 single-positive NK cells and NKG2C^+^ NK cells. We found that the proportion of strong expansion of NKG2C+ NK cells (>20% of all NK cells) or strong expansion of KIR2DL2/L3 single-positive/NKG2C+ NK cells (>45% of all NKG2C^+^ cells) (41) in KIR CenA/A and CenA/B were comparable (day 30, *p* = 0.3309, CenA/A = 6.85%, CenA/B = 0.00%; day 90, *p* = 0.4838, CenA/A = 28.74%, CenA/B = 20.00%; day 180, *p* = 0.6678, CenA/A = 34.21%, CenA/B = 40.00%).

Furthermore, the proportion of KIR2DL2/L3 single-positive/NKG2C^+^ NK cells among the NKG2C^+^ NK cells and the proportion of NKG2C^+^ NK cells among the NK cells were comparable between groups CenA/A and CenA/B on day 30 (*p* = 0.656, *p* = 0.386), 90 (*p* = 0.312, *p* = 0.841), and 180 (*p* = 0.074, *p* = 0.585), while in CMV-reactivated individuals, on day 180, the proportion of KIR2DL2/L3 single-positive/NKG2C^+^ NK cells among the NKG2C^+^ NK cells in the CenA/B group was higher than that in the CenA/A group (CENA/A: 23.92 ± 2.40; CENA/B: 36.32 ± 5.89, *p* = 0.031), indicating that HCMV infection promoted KIR2DL3 single-positive/NKG2C+ NK cell expansion in the CenA/B group.

### An Increased Proportion of KIR2DL2/L3 Single-Positive/NKG2C^+^ NK Cells Correlated With Higher Incidence of aGVHD

The detailed associations among the KIR2DL2/L3 single-positive/NKG2C^+^ proportion, HCMV infection type (no refractory reactivation and refractory reactivation), clinical outcomes (no relapse and relapse, death, or survival), and duration of viremia are presented in **Table 1**. According to the median proportion of KIR2DL2/L3 single-positive/NKG2C^+^ NK cells among the NKG2C^+^ cells, we divided the recipients into a strong KIR2DL2/L3 single-positive/NKG2C^+^ expansion group (>median, day 30 = 10.81%, day 90 = 18.31%, day 180 = 21.19%) and a weak KIR2DL2/L3 single-positive/NKG2C^+^ expansion group (<median). No differences were found in the occurrence of refractory reactivation, cumulative incidence of relapse or overall survival between patients with strong and weak KIR2DL2/L3 single-positive/NKG2C^+^ expansion (data not shown).

**Figure 4:**
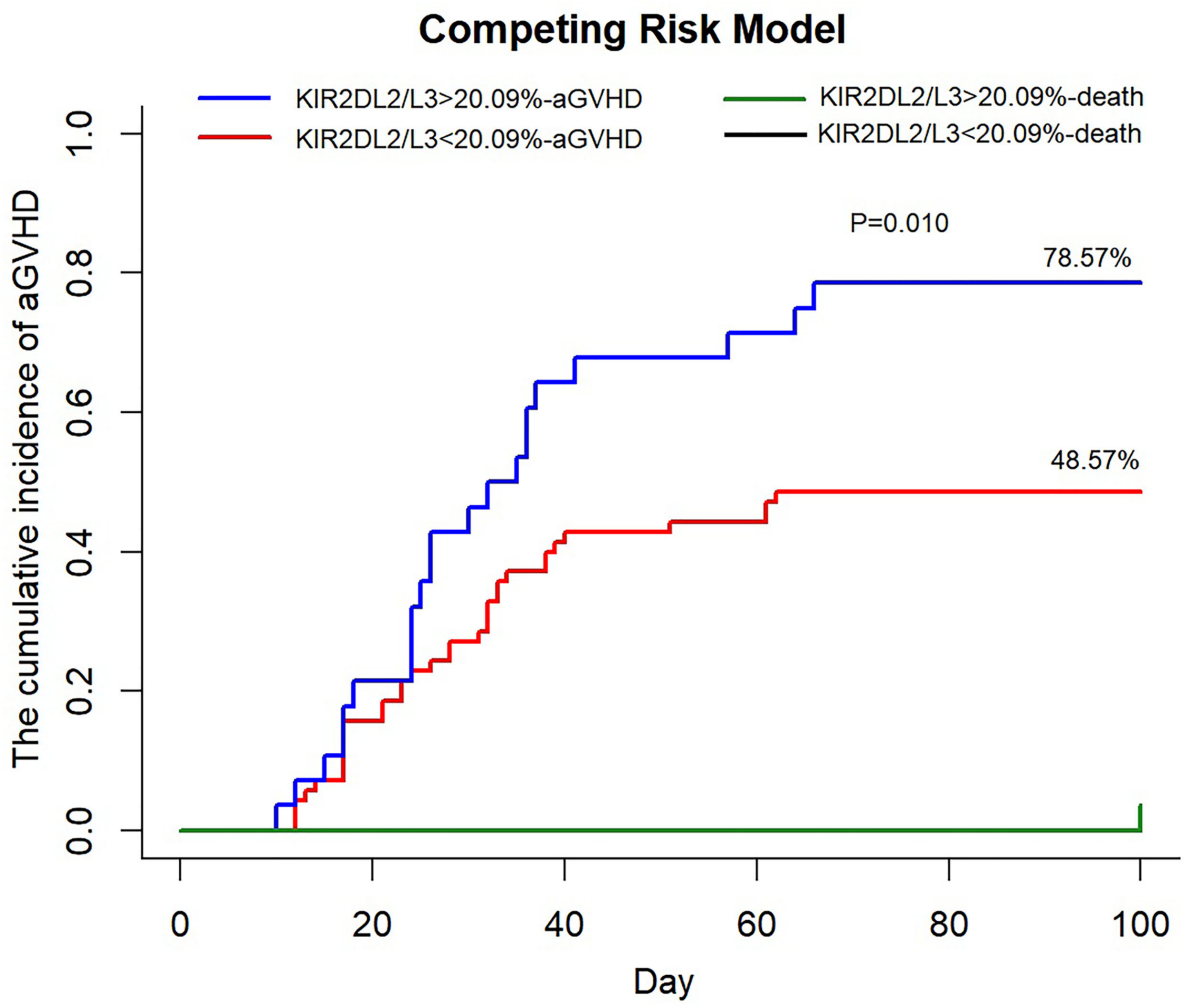
An increased proportion of KIR2DL2/L3 single-positive/NKG2C+ NK cells correlated with higher incidence of aGVHD. KIR2DL2/L3sp%, the proportion of KIR2DL2/L3 single-positive/NKG2C+ NK cells among NKG2C+ NK cells; aGVHD, acute graft-versus-host disease. In competing the risk model, 100 days cumulative incidence of aGVHD in the cohort (KIR2DL2/L3 single-positive/NKG2C+ NK cells < 20.09%, *n* = 69, red line; KIR2DL2/L3 single-positive/NKG2C+ NK cells > 20.09%, *n* = 27, blue line) after HSCT and 100 days cumulative incidence of death, considered as competing risk event (KIR2DL2/L3 single-positive/NKG2C+ NK cells < 20.09%, *n* = 1, black line; KIR2DL2/L3 single-positive/NKG2C+ NK cells > 20.09%, *n* = 1, green line).

We divided the recipients into a strong group (>cutoff value = 20.09%) and a weak group (<cutoff value) based on the proportion of KIR2DL2/L3 single-positive/NKG2C^+^ NK cells at day 30. In univariate analysis, including the strong group of KIR2DL2/L3 single-positive/NKG2C+ NK expansion, CMV-reactivated status, NKG2C genotype, KIR type, recipient’s age, donor’s age, recipient’s gender, donor’s gender, diagnosis, and blood type matching, we found the strong group of KIR2DL2/L3 single-positive/NKG2C+ NK expansion (*p* = 0.006), recipient’s gender (*p* = 0.083), and diagnosis (*p* = 0.050) with *p* < 0.10, which were included in the multivariate analysis. The multivariate analysis showed that the strong group of KIR2DL2/L3 single-positive/NKG2C+ NK expansion affected the incidence of aGVHD (*p* = 0.019). In the competing risk model, we observed that the cumulative incidence of aGVHD of the strong group was greater than the weak group (*p* = 0.010), including death as the competing risk event (**Figure 4**). For the strong group and weak group, 100-day cumulative incidence of aGVHD was 78.57% versus 48.57% in the cohort. It suggested that the expansion of KIR2DL2/L3 played a negative role in preventing aGVHD, which could be exacerbated by CMV infection.

## Discussion

We creatively studied KIR single-positive NK cells to explore the effect of HLA-KIR on NKG2C^+^ expansion and NKG2C^+^ NK cell education after HSCT. Our results first demonstrated that KIR2DL2/L3 single-positive/NKG2C^+^ cells expanded among the NKG2C^+^ NK cells. Additionally, we found that the expression of NKp30, an activating receptor, on KIR2DL2/L3 single-positive/NKG2C^+^ cells was significantly increased compared with that on KIR2DL1 and KIR3DL1 single-positive/NKG2C^+^ cells. We found that the expanded KIR2DL2/L3^+^/NKG2C^+^ NK cells played a negative role in preventing aGVHD.

HLA-C1/C1+ donors had high frequencies of NK cells expressing KIR2DL3 as their only inhibitory KIR and HLA-C2/C2+ donors had high frequencies of NK cells expressing KIR2DL1 as their only inhibitory KIR, suggesting that educating KIR-HLA interactions can achieve this skewing (56). A study of a cohort of HCMV^+^ patients revealed that the expression of KIR2DL1 on NKG2C^+^ NK cells led to significantly larger clonal expansions than KIR2DL2 (41). Another cohort of patients with HCMV reactivation was found to have an expansion of NKG2C^+^ NK cells accompanied by co-expression of KIR with a strong bias towards KIR2DL2/L3 (43). The above studies examined the expression of KIRs on NKG2C^+^ NK cells in HCMV^+^ patients without excluding the interference of other KIRs. However, our research assessed KIR single-positive NK cells in the setting of patients after HSCT. We also uniquely studied its correlation with the expansion of NKG2C^+^ NK cells and clinical outcomes and found that an increased proportion of KIR2DL2/L3 single-positive/NKG2C^+^ NK cells was related to NKG2C^+^ NK cell expansion but not to clinical outcomes.

The surface density of NKp30 correlates with the ability of NK cells to kill target tumor cells and infected cells (57). Our previous study also demonstrated that blocking DNAM-1 and NKp30 substantially decreased NK cell function and NKp30 in combination with DNAM-1 to enhance NK cell hyperresponsiveness (58). This research showed that KIR2DL2/3 single-positive/NKG2C^+^ NK cells expressed higher levels of NKp30, illustrating that expanded KIR2DL2/L3 single-positive/NKG2C^+^ NK cells might have greater cell activity and a more potent function than other NKG2C^+^ NK cell subsets. Moreover, NKp30 mediates cell–cell contact, which induces dendritic cell (DC) activation and the elimination of immature DCs and promotes the production of cytokines, contributing to immune homeostasis (57, 59).

However, in both the whole cohort and HCMV-reactivating individuals, our results demonstrate that three KIR single-positive NK cells show comparable reactivity in group 1 (C1C1), group 2 (C1C1BW4), and group 3 (C1C2BW4). When both donors and recipients carried HLA-C1, the self-single KIR^+^/NKG2C^+^ NK and non-self-single KIR^+^/NKG2C^+^ NK cells showed comparable functions (60). In contrast to a previous study, NKG2C^+^ single self-KIR^+^ (the ligands for their inhibitory KIR were present in the recipient) NK cells were significantly more potent at producing IFN-γ than NKG2C+ single non-self-KIR^+^ NK cells following antiviral therapy for 2 weeks (43). Zahra Kiani and his colleagues found a higher secretion of IFN-γ cell-activated single KIR2DL2-positive NK cells from educated C1 carriers than from uneducated C2/C2 carriers (61). The reason for the different results might be because we studied the cohort at different times after HSCT. Additionally, all three single-positive/NKG2C^+^ NK cells expressed NKG2C, an activating receptor, which can result in comparable cell function.

A recent study reported that KIR2DL3^+^/KIR2DS2^+^ recipients, with a significantly decreased risk of HCMV viremia, only had HLA‐C1 antigens in both the recipients and donors (62). In our cohort, the proportion of KIR2DL2/L3 single-positive NK cells among NKG2C^+^ NK cells also significantly expanded compared to the conventional NKG2C^-^ NK cells. The reason for this could be that a majority of patients after HSCT in that cohort had HCMV reactivation, which could promote the expression of HLA-C1, enhancing the expansion of NKG2C^+^ NK cells.

KIR Cen A/A typing was irrelevant to the higher expansion of KIR2DL2/L3 single-positive/NKG2C^+^ NK cells. Although another study illustrated that strong NKG2C expansion always co-expressed KIR from CenA haplotypes, in which most of the cases recruited KIR2DL1 rather than KIR2DL2/L3, there were still differences. The study and analysis were performed in HCMV^+^ healthy individuals and focused only on the KIR expression and not on single KIR expression. Moreover, the conclusion regarding CenA was derived from the comparison between the CenA/A and Cen B/B groups, and they found no significant differences between the CenA/A and CenA/B groups, confirming our limitation that our cohort did not include CenB/B individuals (41). In addition, CEN A/B also included patients expressing KIR2DS2, and it was our fault that we did not separately mark KIR2DS2 to distinguish it from KIR2DL2/L3.

Moreover, we found that HLA-BW4 did not stimulate the expansion of KIR3DL1 single-positive/NKG2C^+^ cells. The proportion of KIR3DL1 single-positive/NKG2C^+^ cells was significantly lower than that of KIR2DL1 and KIR2DL2/L3 cells on days 90 and 180 in group 3 (C1C2BW4). Van der Ploeg and her colleagues also found that in HSCT patients, HLA-Bw4 could interact with KIR3DL1 with weak affinity, and different allotypes had different inhibitory capacities on KIR3DL1^+^ NK cells (63). Braud and his colleagues also found that the leader sequence peptide of HLA-B is not capable of binding to HLA-E, so it could not be presented to NKG2C and activate NKG2C^+^ NK cells, without the expansion of KIR2DL2/L3 single-positive/NKG2C^+^ NK cells (21). In addition, in HCMV-infected individuals, the fact that CD57^+^NKG2C^+^ NK cells preferentially lack expression of the KIR3DL1 receptor in individuals expressing its HLA-Bw4 ligand can also explain this finding (41).

We found, in agreement with previous studies (64), that the expansion of KIR2DL2/L3 played a negative role in preventing aGVHD, which was more significant under CMV infection. The reason might be that the expanded KIR2DL2/L3+/NKG2C+ NK cells had impaired function to positively modulate the alloreactivity of T cells in GVHD (65–67).

Our research also had limitations. Specifically, the study was limited by the lack of a group with donor–recipient C2C2 pairs, resulting in the unavailability of an analysis of the HLA-C2/KIR2DL1 interaction.

In conclusion, the interaction between KIR and HLA-C could modulate the reconstitution of NKG2C^+^ NK cells, characterized by KIR2DL2/L3 single-positive/NKG2C^+^ NK cell expansion, with increased NKp30 expression, dominated by a combination of KIR2DL2/L3 and HLA-C1. The expansion of KIR2DL2/L3 single-positive/NKG2C^+^ NK cells correlated with higher incidence of aGVHD after HSCT. These data support a novel hypothesis that KIR2DL2/L3 interacts with HLA-C1 between donors and recipients and plays an important role in promoting the expansion of NKG2C^+^ NK cells after HSCT, especially when HCMV is reactivated. However, the underlying mechanism needs to be explored further.

## Data Availability Statement

The raw data supporting the conclusions of this article will be made available by the authors, without undue reservation.

## Ethics Statement

All patients and donors provided written informed consent, and the Ethics Committee of Peking University People’s Hospital approved the study, which was registered at www.clinicaltrials.gov (NCT02978274).

## Author Contributions

X-YZ conceived and designed the study and wrote the manuscript. WZ analyzed and interpreted the data and wrote the manuscript. X-XY and XFL conducted the *in vitro* experiments. Y-JC, X-SZ, YW, L-PX, K-YL, X-HZ, and X-JH performed the clinical examination. All authors contributed to the article and approved the submitted version.

## Funding

This study was supported by grants from the National Natural Science Foundation of China (Grant Nos. 81670166, 81530046, 81870140, 82070184, and 81370666), Innovative Research Groups of the National Natural Science Foundation of China (81621001), the National Key Research and Development Program of China (2017YFA0104500), the Beijing Municipal Science & Technology Commission (Z171100001017098), and Peking University People’s Hospital Research and Development Funds (No. RDL2021-01).

## Conflict of Interest

The authors declare that the research was conducted in the absence of any commercial or financial relationships that could be construed as a potential conflict of interest.

## Publisher’s Note

All claims expressed in this article are solely those of the authors and do not necessarily represent those of their affiliated organizations, or those of the publisher, the editors and the reviewers. Any product that may be evaluated in this article, or claim that may be made by its manufacturer, is not guaranteed or endorsed by the publisher.

## Supplementary Material

Supplementary Figure 1 Representative flow cytometry gating strategy for single-positive KIR/NKG2C^+^ NK cells from lymphocytes in human PBMCs. CD158b, KIR2DL2/L3; CD158a, KIR2DL1; CD158e, KIR3DL1. The representative raw flow plots show both the gating data for KIR2DL1 single positive, KIR2DL2/L3 single positive, and KIR3DL1 single positive NKG2C^+^/NK cells from patients in the general cohort.

Supplementary Figure 2 Representative flow cytometry gating strategy for single-positive KIR/NKG2C^-^NK cells from lymphocytes in human PBMCs. CD158b, KIR2DL2/L3; CD158a, KIR2DL1; CD158e, KIR3DL1. The representative raw flow plots show both the gating data for KIR2DL1 single positive, KIR2DL2/L3 single positive, and KIR3DL1 single positive NKG2C^-^/NK cells from patients in the general cohort.

Supplementary Figure 3 KIR2DL1, KIR2DL2/L3 or KIR3DL1 single-positive NK cells showed comparable reactivity. sKIR2DL1+, single KIR2DL1 positive; sKIR2DL2/L3+, single KIR2DL2/L3 positive; sKIR3DL1+, single KIR3DL1 positive. The cytotoxicity test used cryopreserved PBMCs samples of patients. About 24 h later, 2 million freshly thawed PBMCs were washed twice and transferred to a 96-well round-bottom plate (Corning). In RPMI 1640 supplemented with 10% foetal calf serum with 1000 IU/ml interleukin 2 for 10-14 h, PBMCs were cultured for both spontaneous and IL-2-stimulated NK cytotoxicity assays against K562. **(A–D)** Expression of CD107a and IFN-γ against K562 by KIR2DL2/L3 single NK cells and KIR2DL1 single-positive NK cells from patients with available data after HSCT in group 1 (Day 30, n=7; Day 90, n=7), group 2 (Day 30, n=5; Day 90, n=4) and group 3 (Day 30, n=6; Day 90, n=4) on Day 30 and Day 90. Each plot (triangle, circle or square) shows one patient with indicated receptor expression, middle horizontal lines show mean values for each group, the upper and lower horizontal lines show standard error of mean.**p <* 0.05; ***p <* 0.01; ****p <* 0.001; *****p <* 0.0001; n.s: not significant. (Kruskal–Wallis H test).

Supplementary Figure 4 KIR2DL2/L3 single-positive/NKG2C^+^ NK cells with the *NKG2C^wt/del^* and *NKG2C^wt/wt^* genotypes were significantly expanded. The surface markers of peripheral blood mononuclear cells (PBMCs) from patients’ fresh blood sample were analyzed by flow cytometry, performed in PBS at 4°C for 30 min following Fc blockade.Donor DNA was isolated from total blood using the Puregene BloodCore kit B and *NKG2C* zygosity was assessed. **(A–C)** The proportion of KIR2DL1, KIR2DL2/L3 and KIR3DL1 single-positive/NKG2C^+^ NK cells among the NKG2C+ NK cells from patients accepting *NKG2C^wt/wt^* and *NKG2C^wt/del^* donor cells with available data on Days 30 (*NKG2C^wt/wt^*: n=64; *NKG2C^wt/del^*: n=31), 90 (*NKG2C^wt/wt^*: n=72, *NKG2C^wt/del^*: n=43) and 180 (*NKG2C^wt/wt^*: n=66, *NKG2C^wt/del^*: n=38) (Kruskal–Wallis H test). **(D)** The percentage of KIR2DL2/L3 single-positive/NKG2C^+^ NK cells among the NKG2C^+^ NK cells in HCMV reactivating individuals in the *NKG2C^wt/del^* group was comparable with that in the *NKG2C^wt/wt^* group on Day 90 (P=0.226; *NKG2C^wt/del^, n=38; NKG2C^wt/wt^, n=57*) and Day 180 (P=0.188; *NKG2C^wt/del^, n=36; NKG2C^wt/wt^, n=50*) (Mann–Whitney U test). Each plot (triangle, circle or square) represents one patient with the indicated receptor expression, middle horizontal lines show mean values for each group, and upper and lower horizontal lines show the standard error of the mean. **p <* 0.05; ***p <* 0.01; ****p <* 0.001; *****p <* 0.0001; n.s: not significant.

Supplementary Figure 5 Flow chart of cohort enrolment. Among the 128 patients, NK reconstitution was followed up in 98 individuals on Day 30, 118 individuals on Day 90 and 107 individuals on Day 180.

Supplementary Figure 6 Summary scheme for working model. HSCT, Hematopoietic Stem Cell Transplantation; CMV, cytomegalovirus. Before HSCT, we will test the CMV infection, HLA genotype of both patients and donors, as well as NKG2C genotype and KIR type (CenA/B) of donors. At day 30, day 90 and day 180 after HSCT, we collected the patients’ peripheral blood samples at weekly follow-up exam.We did surface marker staining of peripheral blood mononuclear cells (PBMC) using fresh blood samples and rest PBMC samples will be cryopreserved for cytotoxicity assays later. We divided patients into Group 1, Group 2 and Group 3, according to the patient-donor HLA-C matching, where donor and recipient were both C1C1 in group 1, C1C1BW4 in group 2, and C1C2BW4 in group 3. Besides, according to the number of KIR ligands shared by donors and recipients, we divided patients into groupW, X, Y and Z. We divided the patients into group A to group F according to the types of KIR ligand presented by both donor and recipient.

Supplementary Figure 7 KIR typing was irrelevant regarding the expansion of KIR2DL2/L3 single-positive/NKG2C^+^ NK cells. PB, peripheral blood sample Patient and donor DNA were prepared from the peripheral blood mononuclear cells (PBMCs) for pretransplant HLA typing and stored at -40°C, performed according to instruments.There was no obvious correlation between the proportion of KIR2DL2/L3 single-positive/NKG2C^+^ NK cells among NK cells and the proportion of NKG2C^+^ NK cells among NK cells from patients in the general cohort on Day 30 (n=98), Day 90 (n=118) and Day 180 (n=107). Each circle shows one patient (Spearman rank correlation test). The blue line is the correlation line.

Supplementary Figure 8 An increased proportion of KIR2DL2/L3 single-positive/NKG2C+ NK cells correlated with higher incidence of GVHD. In Kaplan-Meier method, 100 days cumulative incidence of aGVHD in the cohort (KIR2DL2/L3 single positive/NKG2C+ NK cells > 20.09%, n = 69, red line; KIR2DL2/L3 single positive/NKG2C+ NK cells < 20.09%, n = 27, blue line) after HSCT.
