# Supplementary figures and images for: The Interaction of HLA-C1/KIR2DL2/L3 Promoted KIR2DL2/L3 Single-Positive/NKG2C-Positive Natural Killer Cell Reconstitution, Raising the Incidence of aGVHD after Hematopoietic Stem Cell Transplantation
Source: Front Immunol. 2022 Apr 29;13:814334. doi: 10.3389/fimmu.2022.814334 (PMC9101514; doi:10.3389/fimmu.2022.814334)

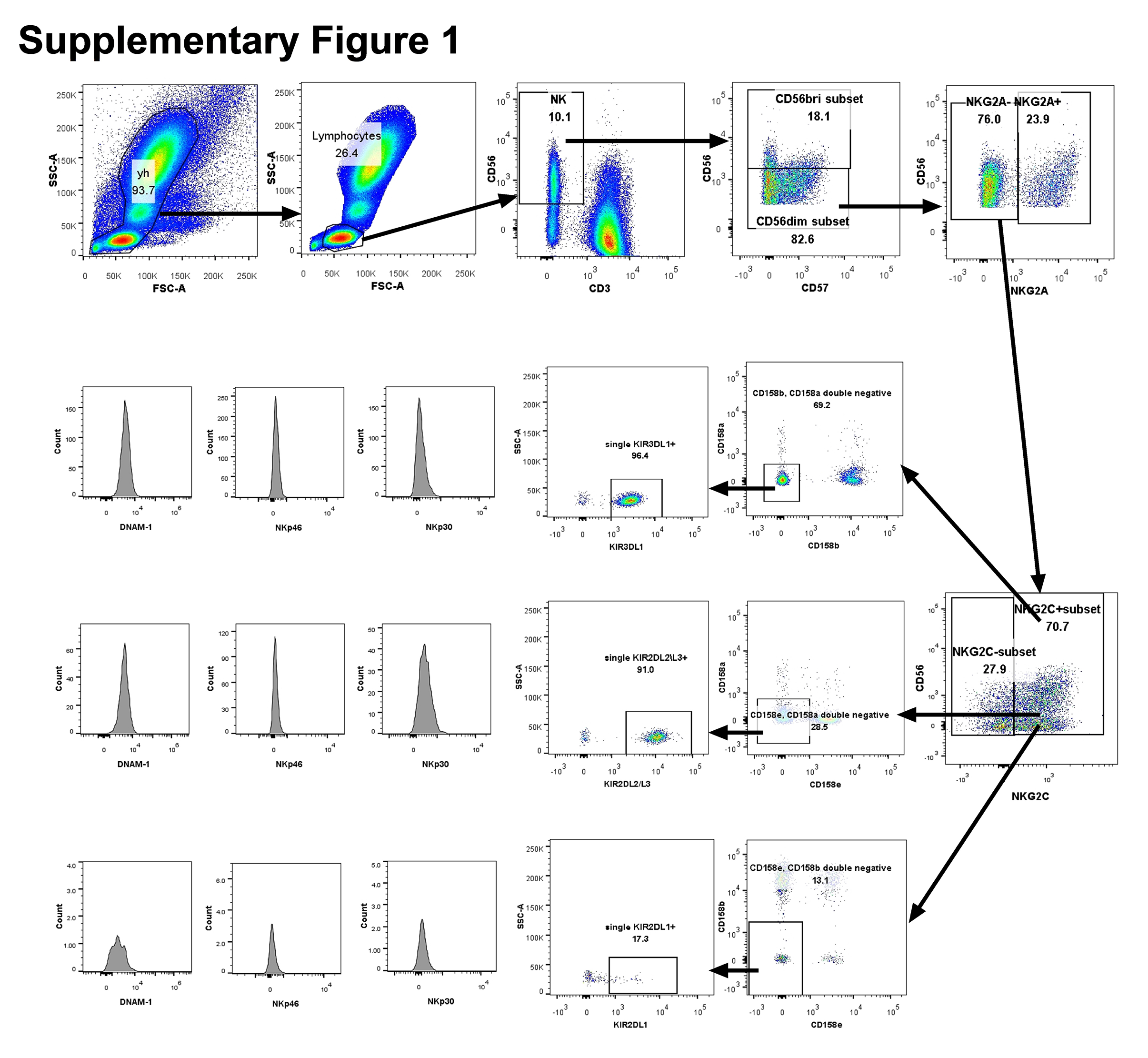

Supplement: Supplementary Figure 1 — Representative flow cytometry gating strategy for single-positive KIR/NKG2C+ NK cells from lymphocytes in human PBMCs. CD158b, KIR2DL2/L3; CD158a, KIR2DL1; CD158e, KIR3DL1. The representative raw flow plots show both the gating data for KIR2DL1 single positive, KIR2DL2/L3 single positive, and KIR3DL1 single positive NKG2C+/NK cells from patients in the general cohort. [file Image_1.tif]

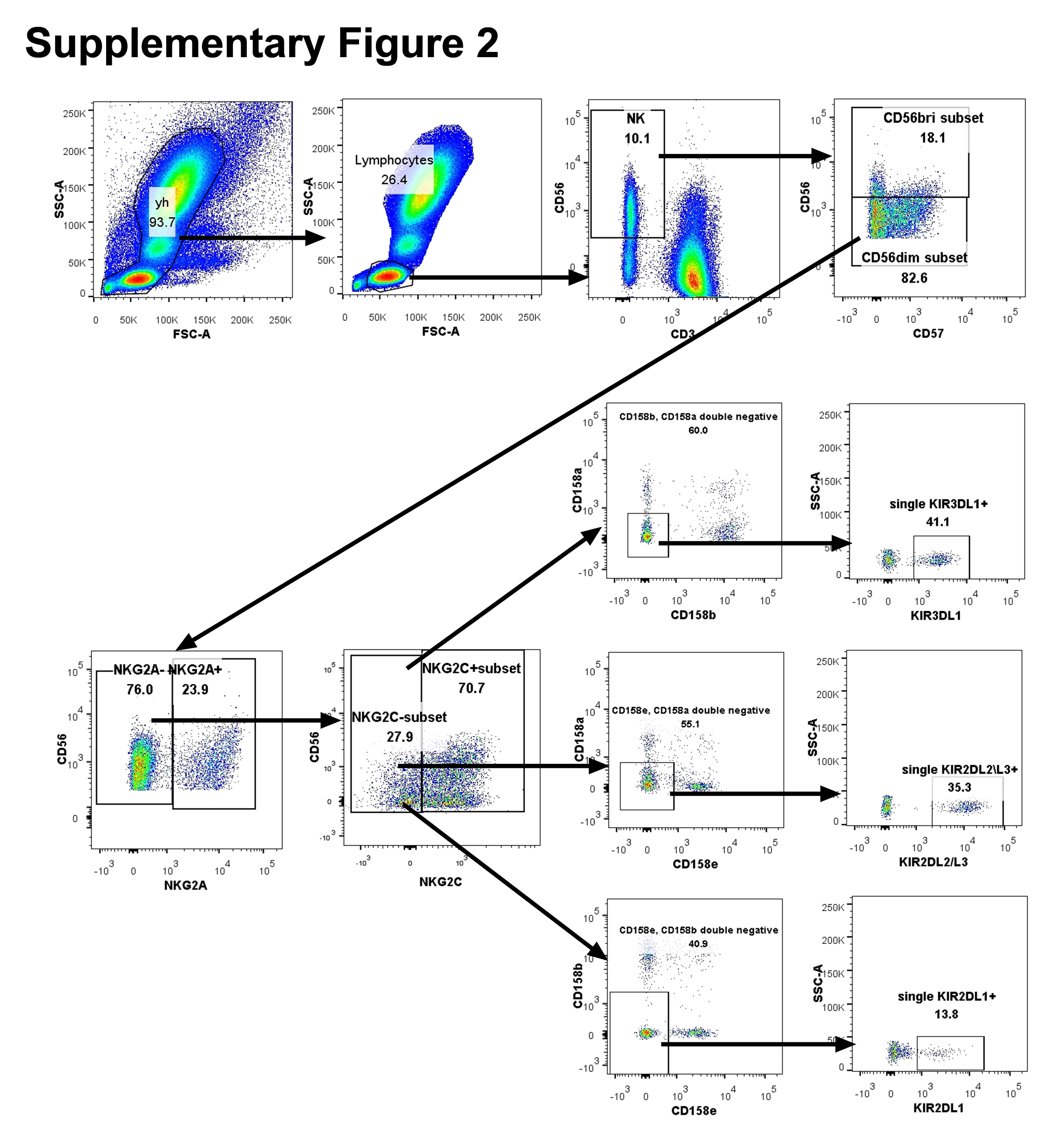

Supplement: Supplementary Figure 2 — Representative flow cytometry gating strategy for single-positive KIR/NKG2C-NK cells from lymphocytes in human PBMCs. CD158b, KIR2DL2/L3; CD158a, KIR2DL1; CD158e, KIR3DL1. The representative raw flow plots show both the gating data for KIR2DL1 single positive, KIR2DL2/L3 single positive, and KIR3DL1 single positive NKG2C-/NK cells from patients in the general cohort. [file Image_2.tif]

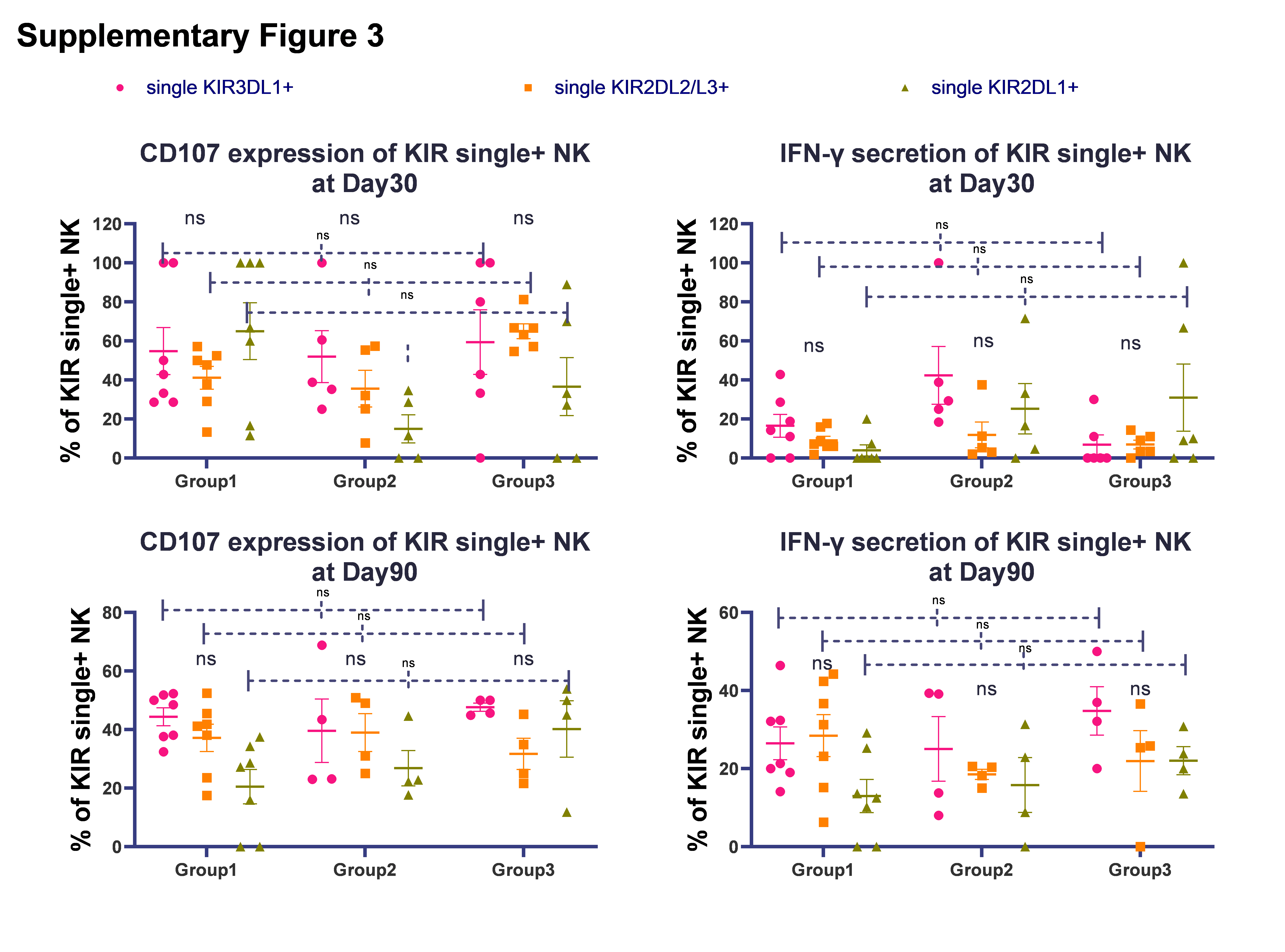

Supplement: Supplementary Figure 3 — KIR2DL1, KIR2DL2/L3 or KIR3DL1 single-positive NK cells showed comparable reactivity. sKIR2DL1+, single KIR2DL1 positive; sKIR2DL2/L3+, single KIR2DL2/L3 positive; sKIR3DL1+, single KIR3DL1 positive. The cytotoxicity test used cryopreserved PBMCs samples of patients. About 24 h later, 2 million freshly thawed PBMCs were washed twice and transferred to a 96-well round-bottom plate (Corning). In RPMI 1640 supplemented with 10% foetal calf serum with 1000 IU/ml interleukin 2 for 10-14 h, PBMCs were cultured for both spontaneous and IL-2-stimulated NK cytotoxicity assays against K562. (A–D) Expression of CD107a and IFN-γ against K562 by KIR2DL2/L3 single NK cells and KIR2DL1 single-positive NK cells from patients with available data after HSCT in group 1 (Day 30, n=7; Day 90, n=7), group 2 (Day 30, n=5; Day 90, n=4) and group 3 (Day 30, n=6; Day 90, n=4) on Day 30 and Day 90. Each plot (triangle, circle or square) shows one patient with indicated receptor expression, middle horizontal lines show mean values for each group, the upper and lower horizontal lines show standard error of mean.*p < 0.05; **p < 0.01; ***p < 0.001; ****p < 0.0001; n.s: not significant. (Kruskal–Wallis H test). [file Image_3.tif]

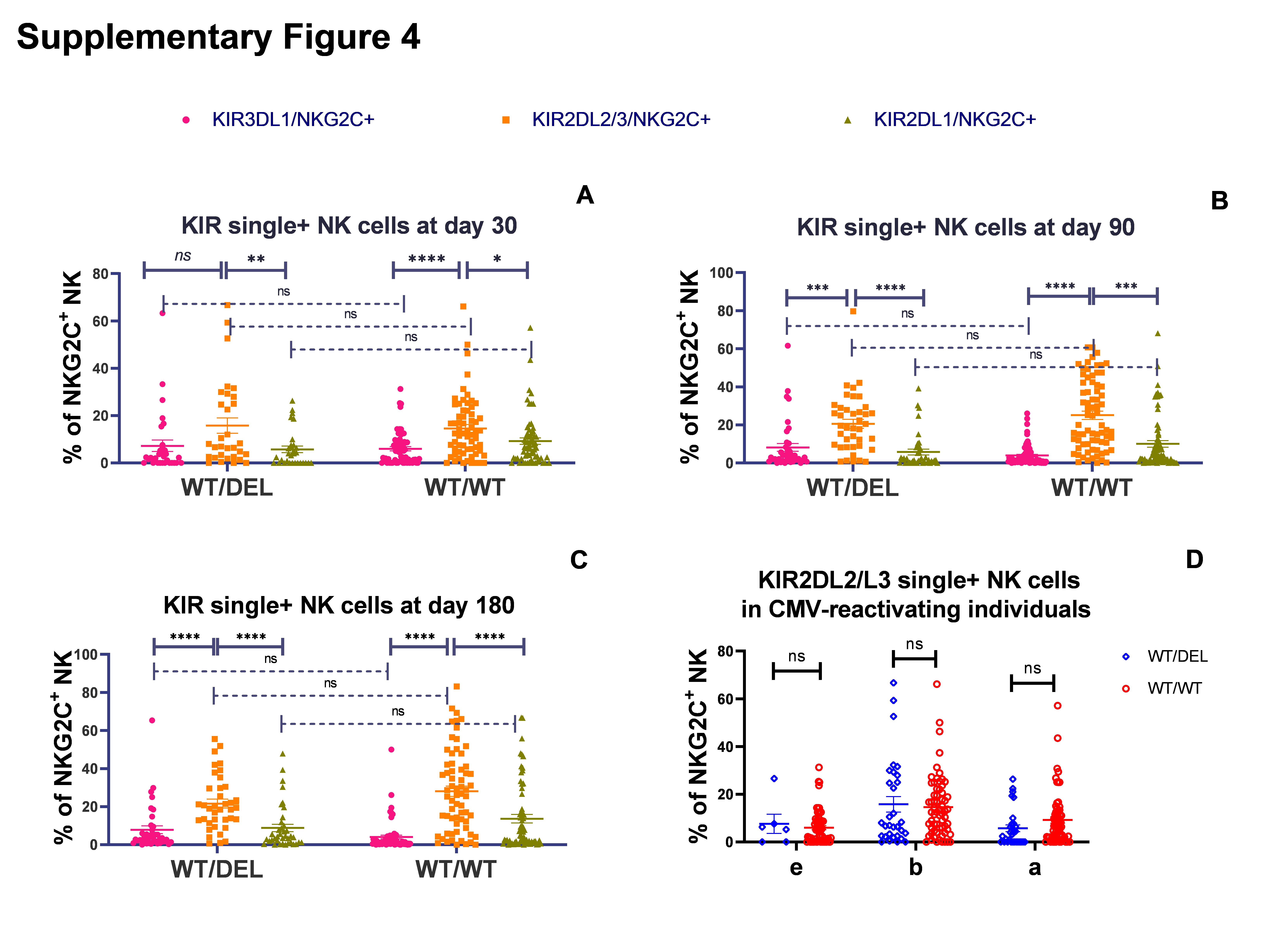

Supplement: Supplementary Figure 4 — KIR2DL2/L3 single-positive/NKG2C+ NK cells with the NKG2Cwt/del and NKG2Cwt/wt genotypes were significantly expanded. The surface markers of peripheral blood mononuclear cells (PBMCs) from patients’ fresh blood sample were analyzed by flow cytometry, performed in PBS at 4°C for 30 min following Fc blockade.Donor DNA was isolated from total blood using the Puregene BloodCore kit B and NKG2C zygosity was assessed. (A–C) The proportion of KIR2DL1, KIR2DL2/L3 and KIR3DL1 single-positive/NKG2C+ NK cells among the NKG2C+ NK cells from patients accepting NKG2Cwt/wt and NKG2Cwt/del donor cells with available data on Days 30 (NKG2Cwt/wt : n=64; NKG2Cwt/del : n=31), 90 (NKG2Cwt/wt : n=72, NKG2Cwt/del : n=43) and 180 (NKG2Cwt/wt : n=66, NKG2Cwt/del : n=38) (Kruskal–Wallis H test). (D) The percentage of KIR2DL2/L3 single-positive/NKG2C+ NK cells among the NKG2C+ NK cells in HCMV reactivating individuals in the NKG2Cwt/del group was comparable with that in the NKG2Cwt/wt group on Day 90 (P=0.226; NKG2Cwt/del, n=38; NKG2Cwt/wt, n=57) and Day 180 (P=0.188; NKG2Cwt/del, n=36; NKG2Cwt/wt, n=50) (Mann–Whitney U test). Each plot (triangle, circle or square) represents one patient with the indicated receptor expression, middle horizontal lines show mean values for each group, and upper and lower horizontal lines show the standard error of the mean. *p < 0.05; **p < 0.01; ***p < 0.001; ****p < 0.0001; n.s: not significant. [file Image_4.tif]

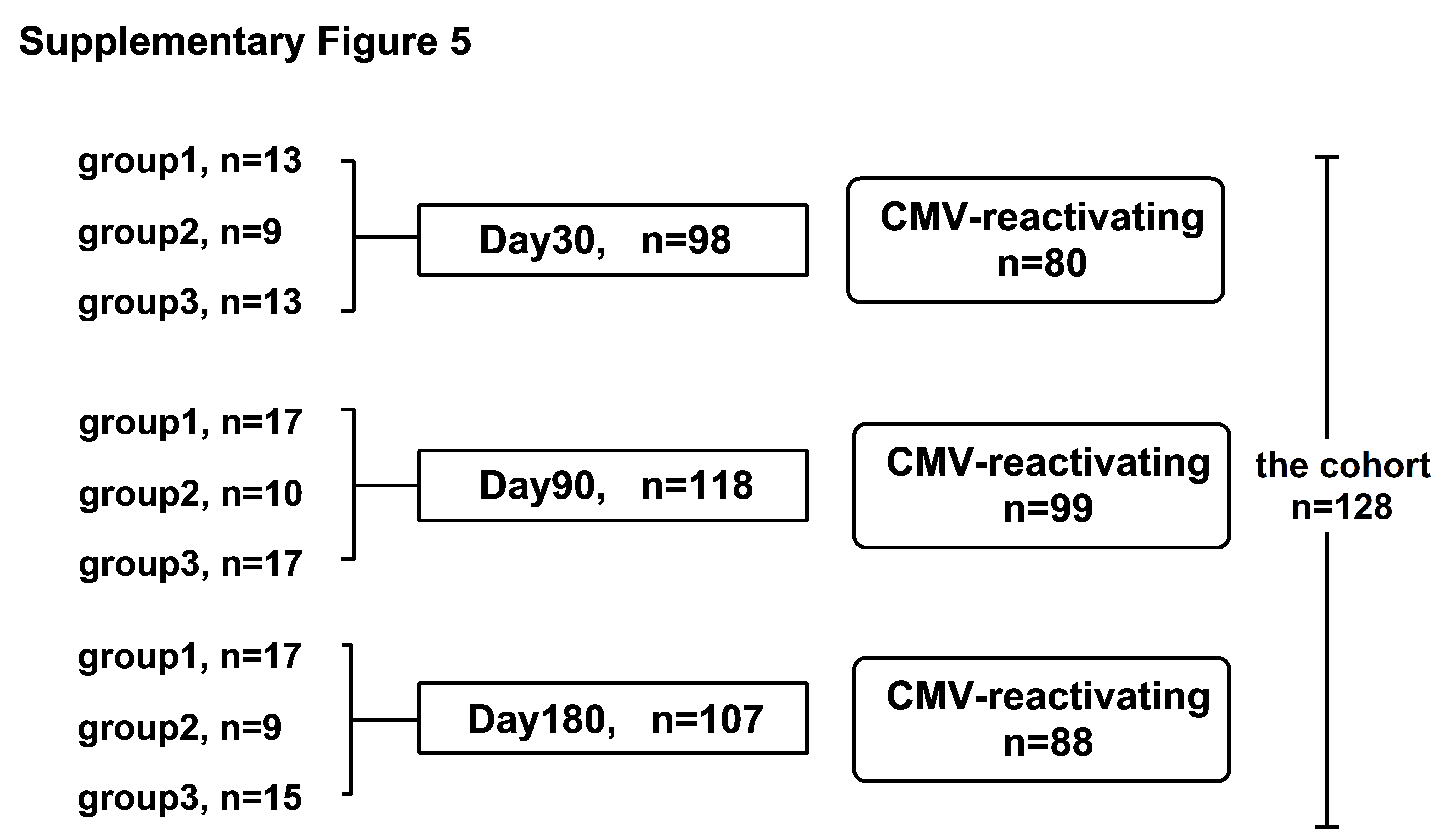

Supplement: Supplementary Figure 5 — Flow chart of cohort enrolment. Among the 128 patients, NK reconstitution was followed up in 98 individuals on Day 30, 118 individuals on Day 90 and 107 individuals on Day 180. [file Image_5.tif]

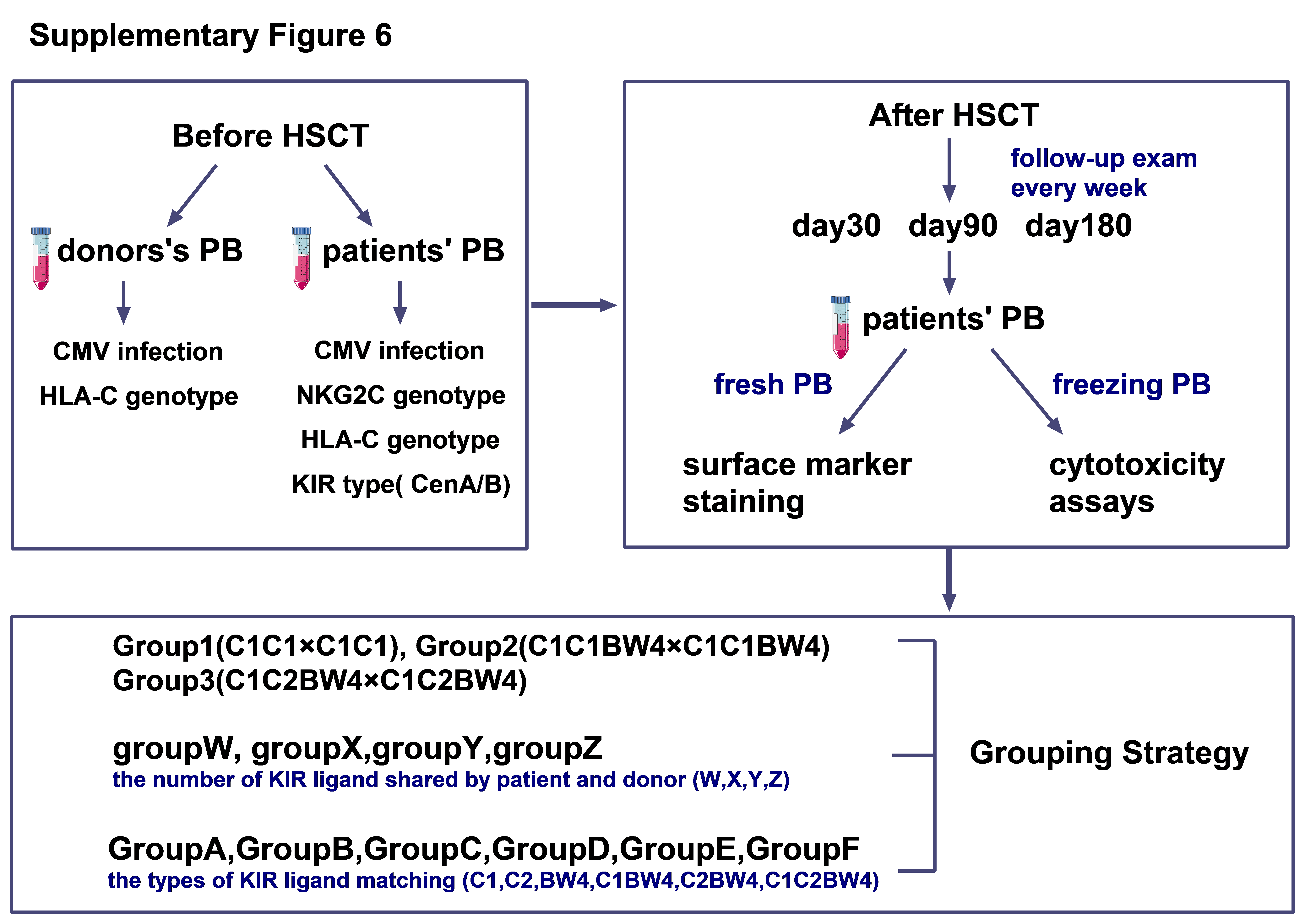

Supplement: Supplementary Figure 6 — Summary scheme for working model. HSCT, Hematopoietic Stem Cell Transplantation; CMV, cytomegalovirus. Before HSCT, we will test the CMV infection, HLA genotype of both patients and donors, as well as NKG2C genotype and KIR type (CenA/B) of donors. At day 30, day 90 and day 180 after HSCT, we collected the patients’ peripheral blood samples at weekly follow-up exam.We did surface marker staining of peripheral blood mononuclear cells (PBMC) using fresh blood samples and rest PBMC samples will be cryopreserved for cytotoxicity assays later. We divided patients into Group 1, Group 2 and Group 3, according to the patient-donor HLA-C matching, where donor and recipient were both C1C1 in group 1, C1C1BW4 in group 2, and C1C2BW4 in group 3. Besides, according to the number of KIR ligands shared by donors and recipients, we divided patients into groupW, X, Y and Z. We divided the patients into group A to group F according to the types of KIR ligand presented by both donor and recipient. [file Image_6.tif]

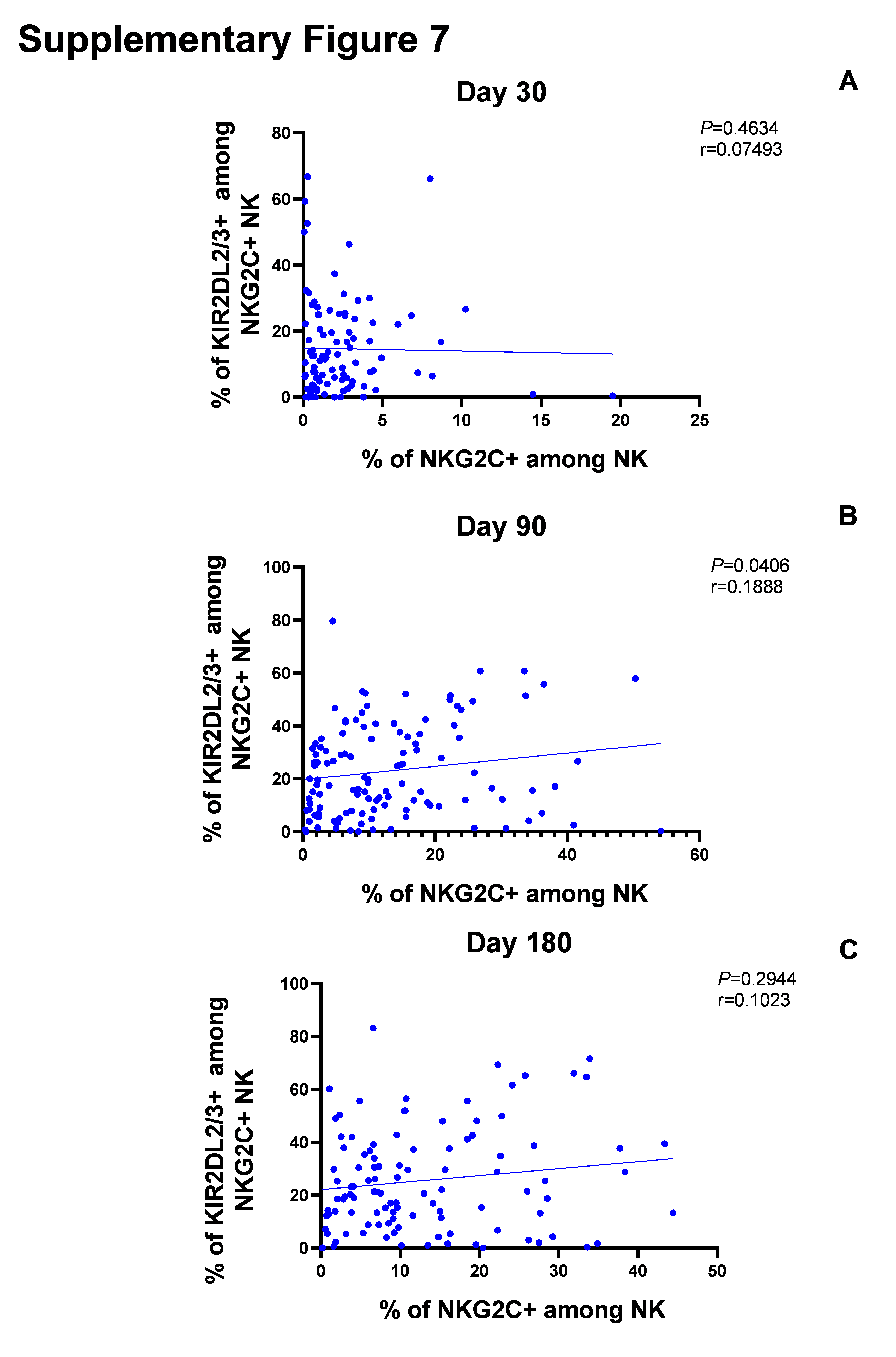

Supplement: Supplementary Figure 7 — KIR typing was irrelevant regarding the expansion of KIR2DL2/L3 single-positive/NKG2C+ NK cells. PB, peripheral blood sample Patient and donor DNA were prepared from the peripheral blood mononuclear cells (PBMCs) for pretransplant HLA typing and stored at -40°C, performed according to instruments.There was no obvious correlation between the proportion of KIR2DL2/L3 single-positive/NKG2C+ NK cells among NK cells and the proportion of NKG2C+ NK cells among NK cells from patients in the general cohort on Day 30 (n=98), Day 90 (n=118) and Day 180 (n=107). Each circle shows one patient (Spearman rank correlation test). The blue line is the correlation line. [file Image_7.tif]

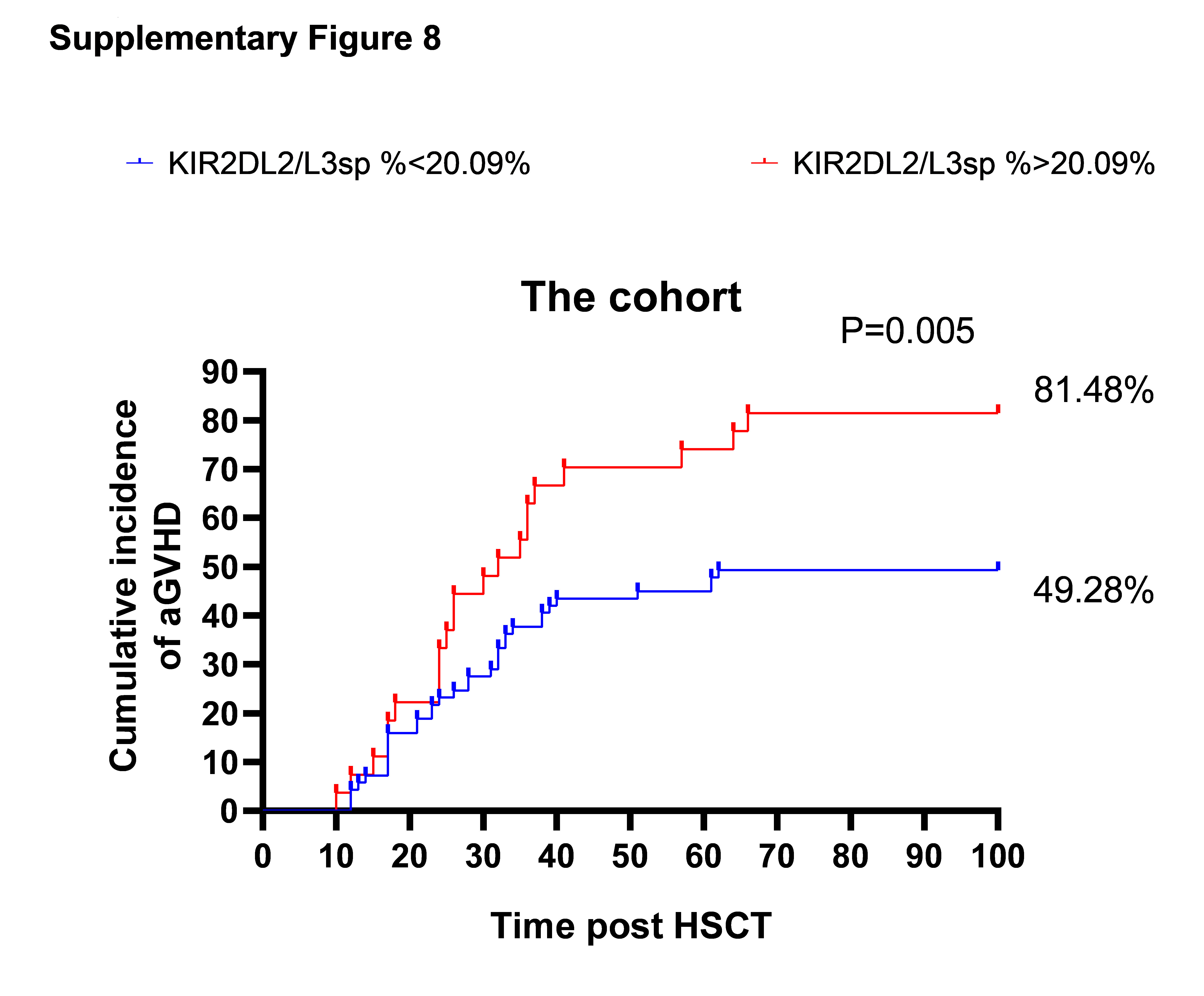

Supplement: Supplementary Figure 8 — An increased proportion of KIR2DL2/L3 single-positive/NKG2C+ NK cells correlated with higher incidence of GVHD. In Kaplan-Meier method, 100 days cumulative incidence of aGVHD in the cohort (KIR2DL2/L3 single positive/NKG2C+ NK cells > 20.09%, n = 69, red line; KIR2DL2/L3 single positive/NKG2C+ NK cells < 20.09%, n = 27, blue line) after HSCT. [file Image_8.tif]
