# Supplementary material for: The Interaction of HLA-C1/KIR2DL2/L3 Promoted KIR2DL2/L3 Single-Positive/NKG2C-Positive Natural Killer Cell Reconstitution, Raising the Incidence of aGVHD after Hematopoietic Stem Cell Transplantation
Source: Front Immunol. 2022 Apr 29;13:814334. doi: 10.3389/fimmu.2022.814334 (PMC9101514; doi:10.3389/fimmu.2022.814334)
Supplement: Supplementary file 9 [file Table_1.docx]

**Supplementary Table 1. Flow cytometry antibody list**

| **No** | **Antibody** | **Format** | **Clone** | **Company** |
| --- | --- | --- | --- | --- |
| 1 | CD25 | BUV395 | 2A3 | BD Biosciences |
| 2 | CD122 | BB515 | Mik-β3 | BD Biosciences |
| 3 | CD94 | Percp-cy5.5 | HP-3D9 | BD Biosciences |
| 4 | CD158e | APC-Vio770 | REA168 | Miltenyi Biotec |
| 5 | CD3 | BV510 | UCHT1 | BD Biosciences |
| 6 | CD56 | BUV737 | NCAM16.2 | BD Biosciences |
| 7 | CD159a(NKG2A) | PE-Vio770 | REA110 | Miltenyi Biotec |
| 8 | CD57 | BV605 | QA17A04 | BioLegend |
| 9 | CD57 | PE-CF594 | NK-1 | BD Biosciences |
| 10 | CD158a | BV421 | HP-3E4 | BD Biosciences |
| 11 | CD158b | PC5.5 | GL183 | Beckman Coulter |
| 12 | CD158b | PE | CH-L | BD Biosciences |
| 13 | CD107a | BV786 | H4A3 | BD Biosciences |
| 14 | IFN-γ | PE | B27 | BD Biosciences |
| 15 | NKP30 | BV605 | AF29–4D12 | BD Biosciences |
| 16 | NKP46 | BV786 | 29A1.4 | BD Biosciences |
| 17 | NKG2D | PerCP-cy5.5 | 1D11 | BD Biosciences |
